# Structure determination and activity manipulation of the turfgrass ABA receptor FePYR1

**DOI:** 10.1038/s41598-017-14101-9

**Published:** 2017-10-25

**Authors:** Zhizhong Ren, Zhen Wang, X. Edward Zhou, Huazhong Shi, Yechun Hong, Minjie Cao, Zhulong Chan, Xue Liu, H. Eric Xu, Jian-Kang Zhu

**Affiliations:** 1 Shanghai Center for Plant Stress Biology and Center for Excellence in Molecular Plant Sciences, Chinese Academy of Sciences, Shanghai, 200032 China; 2 University of Chinese Academy of Sciences (CAS), Shanghai, 200032 P. R. China; 3 Laboratory of Structural Sciences and Laboratory of Structural Biology and Biochemistry, Van Andel Research Institute, Grand Rapids, MI USA; 4 Department of Chemistry and Biochemistry, Texas Tech University, Lubbock, Texas 79409 USA; 5 Key Laboratory of Horticultural Plant Biology, Ministry of Education, College of Horticulture & Forest Sciences, Huazhong Agricultural University, Wuhan, 430070 China; 6 Key Laboratory of Receptor Research, VARI-SIMM Center, Center for Structure and Function of Drug Targets, Shanghai Institute of Materia Medica, Chinese Academy of Sciences, Shanghai, China; 7 Department of Horticulture and Landscape Architecture, Purdue University, West Lafayette, Indiana, 47907 USA

## Abstract

Turfgrass are widely cultivated ornamental plants that have important ecological, societal and economical values. However, many turfgrass species are susceptible to drought and demand frequent irrigation thus consuming large amounts of water. With the ultimate goal of improving drought resistance in turfgrass, we identified several ABA receptors in turfgrass that are important to mediate ABA signaling and drought stress response. The ABA receptor FePYR1 from turfgrass *Festuca elata* was demonstrated to bind ABA as a monomer. Crystal structure analysis revealed that FePYR1 recognizes and binds ABA by the common gate-latch-lock mechanism resembling the Arabidopsis ABA receptors, but the ABA binding pocket in FePYR1 shows discrepant residues resulting in different binding affinity to ABA. Structure-guided alterations of amino acid residues in FePYR1 generated ABA receptor variants with significantly increased ABA binding affinity. Expression of FePYR1 in Arabidopsis conferred enhanced drought resistance in the transgenic plants. These findings provided detailed information about FePYR1 and demonstrated that structure-assisted engineering could create superior ABA receptors for improving plant drought resistance. The detailed structural information of FePYR1 would also assist future rational design of small molecules targeting specific ABA receptors in economically important plant species.

## Introduction

The phytohormone abscisic acid (ABA) is a vital small molecule that plays crucial roles in many physiological and developmental processes in plants such as seed germination, bud dormancy^1^ and adaptive responses to environmental stresses^2^. ABA has been found in all kingdoms of living organisms except Archaea^3^. In plants, one of the main functions of ABA is to modulate water balance and adjust osmotic potential of the cells^2^. Under water deficit conditions, the cellular ABA concentration is increased by releasing ABA from its conjugated forms stored in the vacuole, ER or apoplastic space^4^ as well as via de novo ABA biosynthesis^5^; elevated ABA promotes stomatal closure and inhibits stomatal opening, thus preventing water loss from leaves.

ABA as a signaling molecule mediates physiological responses through dedicated signaling pathways. In the last several decades, many critical components mediating ABA signaling have been identified using genetic, molecular, biochemical and pharmacological studies^6^. However, owing to genetic redundancy of the ABA receptors, how the ABA signal is perceived and transduced has been debated for a long time until a breakthrough in 2009 when a novel family of proteins containing steroidogenic acute regulatory protein (StAR)-related lipid-transfer (START) domain was discovered as ABA receptors^7–11^. In *Arabidopsis*, there are 14 members of such proteins known as Pyrabactin Resistance 1 (PYR1) and PYR1-like 1–13 (PYL1-PYL13)^12^ or Regulatory Component of ABA Receptor (RCAR1-RCAR14)^7^ (herein referred to as PYLs). These members are classified into three subfamilies based on amino acid sequence identity^12^, and members in subfamily III can be considered as dimeric ABA receptors in solution, while members in other two subfamilies are monomeric in solution^10,13^. Intriguingly, recent quantitative data showed that monomeric receptors had stronger ABA-binding affinity than dimeric receptors. For example, the monomeric AtPYL9 in the subfamily I, with a small K_d_ value of 0.66 μM^7^, displayed stronger ABA-binding affinity than the dimeric AtPYL1 and AtPYR1 in the subfamily III^14^. Based on several crystallographic studies, a well-accepted mechanism of ABA recognition by the PYLs has been proposed. ABA signal is perceived and transduced by a so-called gate-latch-lock mechanism, in which ABA binding to the PYL receptor’s ligand binding pocket induces a conformational change leading to closure of the pocket by the gate and latch, which creates an interaction surface for the downstream PP2Cs^8^. ABA-induced interaction of PYLs with PP2Cs releases the inhibitory effect of PP2Cs on SnRK2 protein kinases, resulting in the activation of SnRK2s and phosphorylation of the downstream targets such as ABF transcription factors^15^. The structural details of ABA receptor binding to ABA have been utilized for rational design of bioactive molecules capable of improving drought resistance in crops, which has a great potential to benefit agriculture in the future^16,17^. Interestingly, although the core components involved in ABA signaling were found to be conserved across the plant kingdom, the sequences and numbers of each components show significant variations from aquatic to terrestrial plants^18^. This suggests that different plant species have developed similar but somewhat distinct mechanisms of ABA perception and responses. Thus, further investigation of different plant species could provide novel insights into the structural basis of distinct responses of different plants to ABA, which could further assist structure-based design of bioactive molecules targeting different crops for stress resistance.

Turfgrass is among the most widely planted ornamental plants in the world and serves important functions in soil stabilization, water attenuation and filtration, adjusting local ecosystem and providing safe surfaces for activities in home lawns, sport fields and golf courses. In addition, service grasslands have also been shown to have a beneficial impact on human health^19^. However, maintenance of turfgrass is becoming more challenging due to various environmental stresses. Turfgrass requires extensive irrigation and consumes large amounts of water during dry seasons, which leads to a considerable financial cost, estimated to be €53 billion in 2006 for Europe and $62 billion in the USA in 2000 just for golf courses^20^. Genetic modification and application of plant growth regulators could be strategies of choices to improve drought resistance in turfgrass. In this study, we identified several candidate genes for ABA receptors in the turfgrass *Festuca elata* and *Zoysia japonica*, and then we confirmed that one of the candidate receptors, named FePYR1, functions as an ABA receptor through crystal structure and biochemical analyses. Furthermore, we found that expression of FePYR1 significantly enhances drought resistance in transgenic *Arabidopsis*. Interestingly, structure-guided design of FePYR1 could substantially increase its ABA binding affinity. Our findings suggest that engineering of turfgrass ABA receptors could enhance ABA signaling, with great potential to improve drought stress resistance in turfgrass.

## Results

### Identification of putative ABA receptor genes in turfgrass

**Figure 1:**
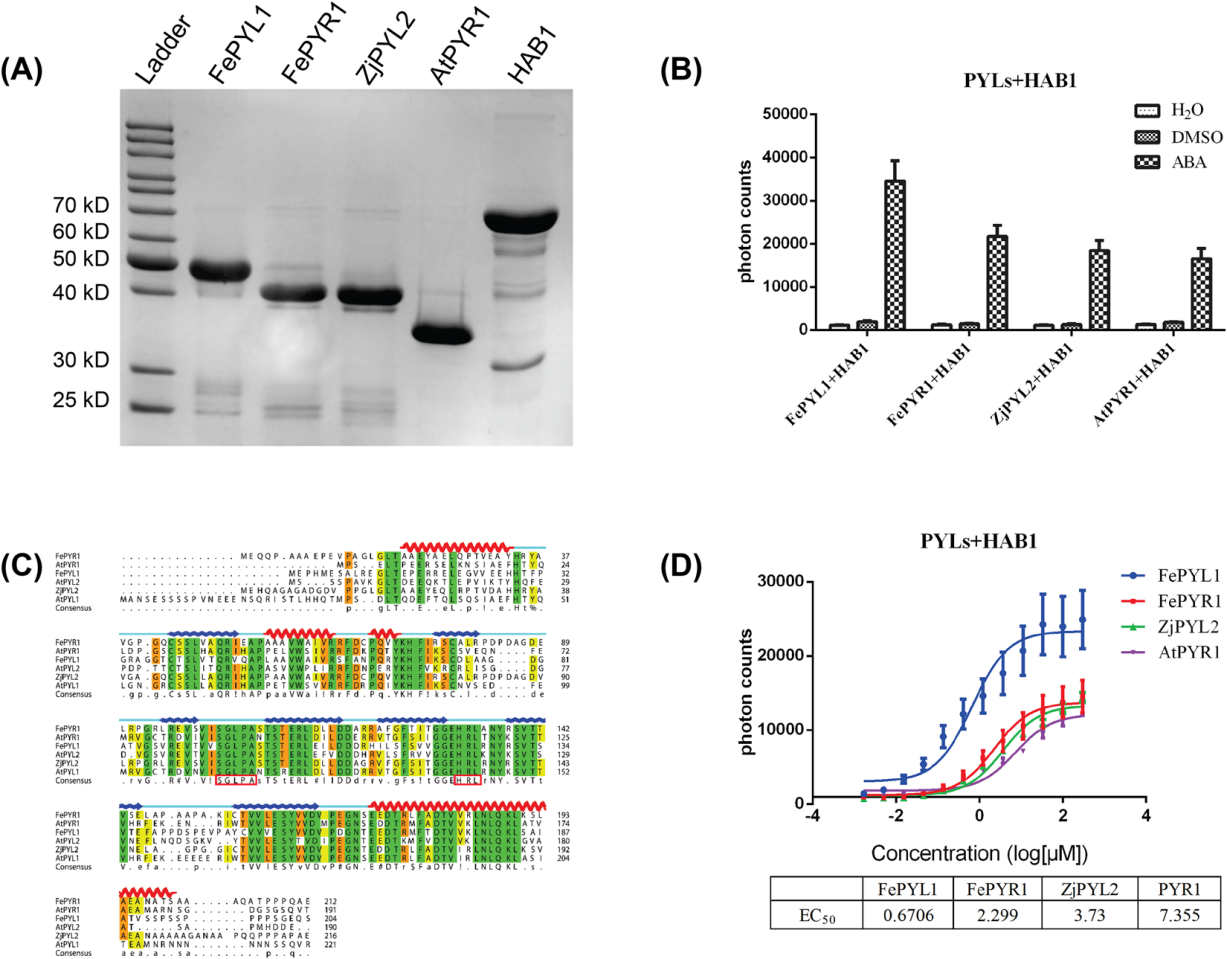
Identification of putative ABA receptors in turfgrass. (**A**) SDS-PAGE gel showing purified H6-SUMO tagged candidate ABA receptor proteins, H6-SUMO-PYR1 and biotin-MBP-HAB1. (**B**) Alpha-Screen assay for the interactions between ABA receptors and AtHAB1 in the presence of 100 μM ( + )-ABA (n = 3, error bars are means ± SD). DMSO and H_2_O were used as negative controls. (**C**) Sequence alignment between the three candidate turfgrass ABA receptors and the subfamily III members of Arabidopsis ABA receptors. The secondary structure elements of FePYR1 were highlighted by wave lines above the alignment. Conserved residues forming the gate and latch are colored in red. (**D**) ABA dose-dependent interactions between AtHAB1 and PYLs as determined by Alpha-Screen assays. The table at the lower panel shows the EC_50_ values for the interactions (n = 3, error bars are means ± SD).

Since the whole genome sequences of turfgrass were not available, we searched for the homologous genes of the ABA receptors in *Festuca elata* and *Zoysia japonica* from the assembled transcriptome sequence data of these two turfgrass species. Total nine ABA receptor candidate genes, named FePYR1, FePYL1-5 and ZjPYL1-3, were identified and the sequence data of these genes were deposited in the Genebank database. The CDS sequences of these candidate genes were synthesized, and the interactions between His-tagged PYLs and biotin-tagged PP2Cs were analyzed by the AlphaScreen binding assay (Fig. S1). Three putative ABA receptors, FePYR1, FePYL1 and ZjPYL2, showed strong interactions with the *Arabidopsis* PP2C enzyme AtHAB1 (Fig. S1B and S1C, Fig. 1A and B). Further sequence alignment suggested that these three putative ABA receptors are homologs of the Subfamily-III members of the *Arabidopsis* ABA receptors^12^, and FePYR1 has the highest identity (51%) with AtPYR1, while FePYL1 and ZjPYL2 show highest identities with AtPYL2 (56% and 52% respectively) (Fig. 1C). The hallmark regions, especially the gate (residues: SGLPA) and latch (residues: HRL) regions of the ABA receptors, are well conserved between the turfgrass and *Arabidopsis*. These results suggest that the three putative ABA receptors may function to mediate ABA signaling in turfgrass. Analysis of dose response revealed that the EC_50_ values of FePYL1, FePYR1, ZjPYL2 in interacting with AtHAB1 are10-fold, 3.2-fold and 1.9-fold higher than that of AtPYR1 (Fig. 1D), which suggests that these three ABA receptors have higher potencies than AtPYR1 in promoting ABA-dependent interactions with AtHAB1. These differences are likely due to structural variations in ABA receptors from different plant species.

### FePYR1 binds ABA and exists as a monomer

**Figure 2:**
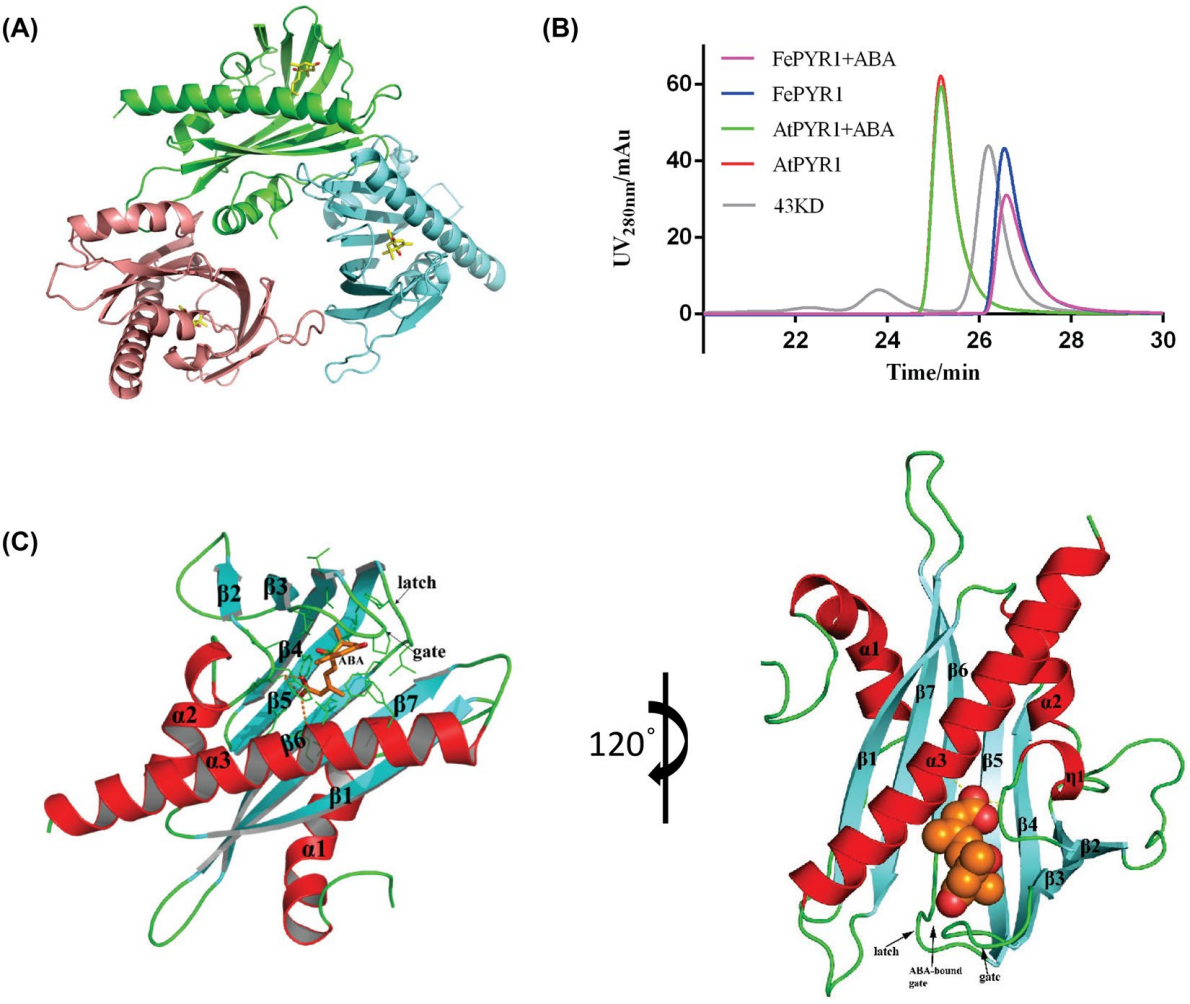
Structure determination of FePYR1-ABA complex. (**A**) The trimeric structure of FePYR1-ABA complex determined in the P4_2_2_1_2 asymmetric unit. The three FePYR1 protomers are shown as ribbon models in green (complex A), cyan (complex B) and pink (complex C), respectively, with ABA as yellow stick models. (**B**) Elution profile of AtPYR1 and FePYR1 with or without (+)-ABA by size-exclusion column chromatography. 43 kD protein marker was used to assess the molecule weight. (**C**) Structure of FePYR1 protomer in complex with (+)-ABA (complex A). The protein structure is differentially colored by secondary structural elements, with helices colored in red, β-strands in cyan, and loops in green. The ABA molecules are shown as aurantia stick and sphere before and after 120° rotation, respectively. The gate and latch loops are indicated by arrows.

We attempted to crystalize the three ABA receptor proteins, and found that only the FePYR1-ABA complex generated high-quality and diffractable crystals (Fig. S2A–2D). The structure of FePYR1-ABA was solved by molecular replacement starting from a model of the *Arabidopsis thaliana* protein AtPYL2 with the statistics of the structure refinement shown in Table S1. The FePYR1-ABA complex crystal structure was determined at a resolution of 2.7 Å, and the overall structure revealed a receptor/ligand complex with 1:1 stoichiometry. The ABA-bound FePYR1 appeared as a trimeric form arranged about 120° from each other in an asymmetric unit of the P4_2_2_1_2 space group (Fig. 2A). To validate whether the FePYR1-ABA complex forms an authentic trimer in solution, we used HPLC to evaluate the molecular weight of FePYR1 with or without ABA. Figure 2B shows that AtPYR1-apo had a peak same as the ABA-bound AtPYR1 with a molecule weight larger than the 43-kD marker, which is consistent with the observation that AtPYR1 assembles into a cis-homodimer with one ABA-bound and one ABA-free subunit^10^, while FePYR1-apo and FePYR1-ABA complex exhibited a molecular weight smaller than the 43-kD marker protein. This result suggests that FePYR1 is a monomeric rather than trimeric ABA receptor in turfgrass which is different from the *Arabidopsis* AtPYR1, and the trimeric arrangement in the P4_2_2_1_2 crystal form is likely due to the crystal packing.

The monomeric FePYR1 structure showed a topology with 7-stranded anti-parallel β-sheet and two short N-terminal α-helix wrapping around a long C-terminal α-helix (Fig. 2C), which represents the characteristic feature of the PYL family of proteins^21^. In the FePYR1-ABA complex structure, ABA with its hydrocarbon chain and cyclohexene ring is centered in the hydrophobic pocket of FePYR1. The ABA binding of FePYR1 also employs a conserved gate (β3–β4) and latch (β5–β6) mechanism, which resembles the PYR1/PYLs in *Arabidopsis*. These structural features of FePYR1 strongly support that FePYR1 is an authentic ABA receptor in the turfgrass species *Festuca elata*.

### Features of FePYR1-ABA binding

**Figure 3:**
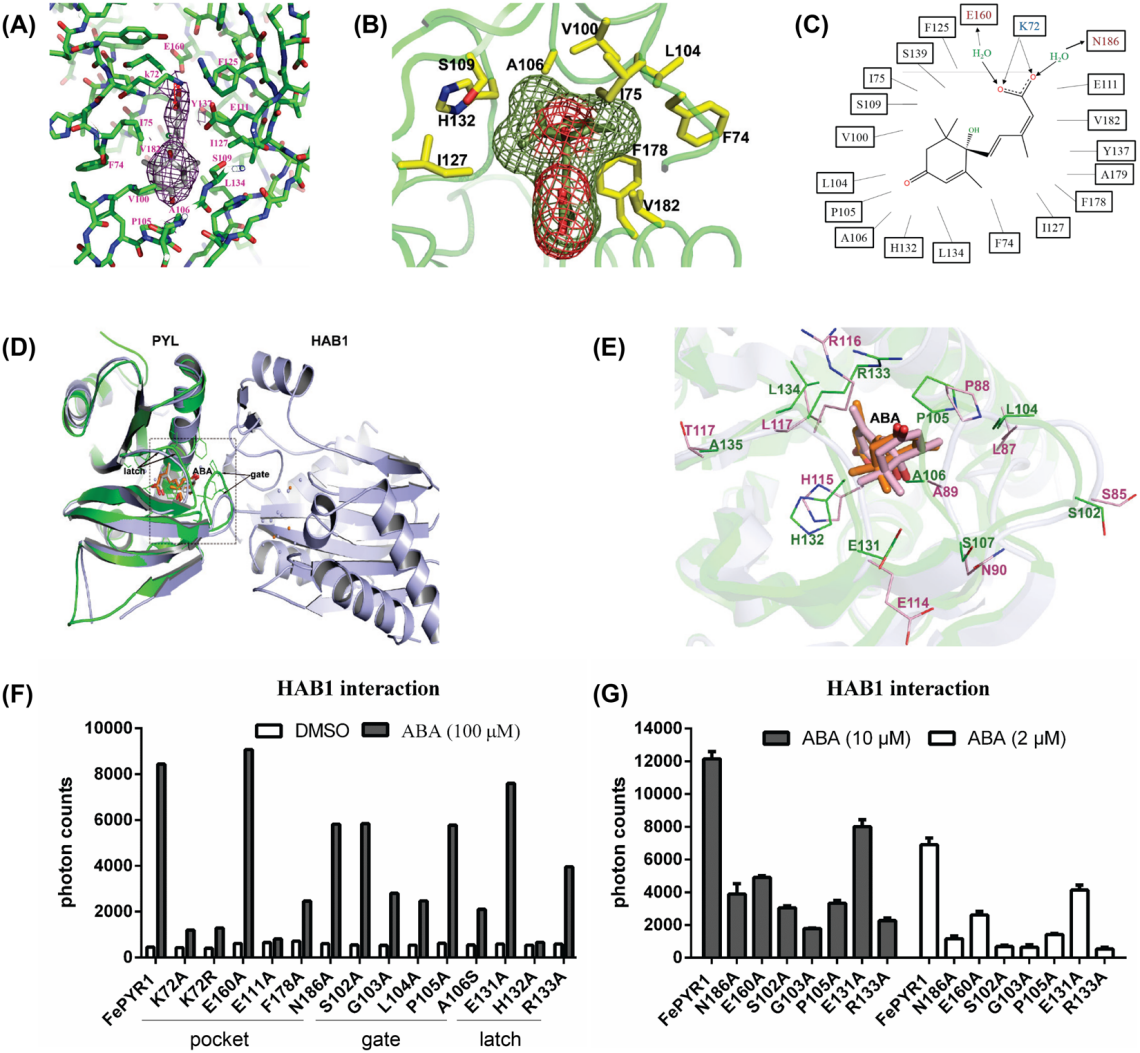
Structural basis of ABA recognition by FePYR1. (**A**) 2Fo-Fc electron density map of ABA and its surrounding residues contoured at 1.0 σ. (**B**) The mono-methyl and the dimethyl groups of ABA are surrounded by stereo-selective residues of FePYR1. (**C**) Schematic presentation of the interactions between bound ABA and FePYR1 binding pocket residues within 4 Å. Charged interactions and hydrogen bonds are indicated by arrows, hydrophobic interactions indicated by solid lines. (**D**,**E**) Overlap of FePYR1 (green) with AtPYR1-HAB1 complex (gray). The differential gate and latch loop of FePYR1 (green) and AtPYR1 complex (gray) (PDB code: 3qn1) are indicated by arrows in (**D**). (**E**) Enlarged interface interacting with AtHAB1 indicated by dash line box in (**D**). (**F**,**G**) Mutations of the key residues in the binding pocket, gate and latch regions of FePYR1 compromise its interactions with AtHAB1 in the presence of ABA at concentrations of 100 μM (**F**), 10 μM and 2 μM ABA (**G**).

The electron density map shows that ABA is tethered in the 428.0 Å^3^ of protein cavity with its hydrocarbon chain and cyclohexene ring fitting closely into the hydrophobic pocket of FePYR1 (Fig. 3A). The inter-molecular interactions between ABA and FePYR1 are diagrammed in Fig. 3C. Each of the three polar groups of ABA forms direct or water-mediated hydrogen bonds with the receptor. The terminal carboxylate of ABA is anchored in the bottom of the cavity of FePYR1 by an interaction between the positively charged inward-pointing K72 side chain with the negatively charged carboxyl group of ABA (Fig. 3C). Based on the structural statistics of FePYR1-ABA and published data^7,8^, E160 and N186 are assumed to form water-mediated hydrogen bonds with the acid head group of ABA (Fig. 3C). Figure 3B shows that the mono-methyl group on the cyclohexene ring fits into a narrow hydrophobic pocket formed by residues F74, I75, V100, L104, F178, and V182, while the dimethyl group fits into a larger pocket formed by A106, S109, I127 and H132. All these interactions including hydrogen bonds and hydrophobic interactions presumably contribute to the stereo-selectivity and ABA binding affinity. At top orientation of the cavity, the carbonyl group on the cyclohexene ring of ABA points to the cavity entrance and is capped by the P105 in the loop (β3–β4) as a gate, and the R133 in the loop (β5–β6) points across the entry as a latch (Fig. 3E). Resembling the *Arabidopsis* ABA receptors, FePYR1 also has a conserved SGLPA gate-like loop and a HRL latch-like loop (Fig. 2C), and these regions form the major part of the binding interface with the PP2C protein. Comparison between the gate-latch like regions of FePYR1 with the interface of AtPYR1 contacting to the co-receptor AtHAB1(PDB code: 3qn1) reveals distinct shifts within the functional loops of FePYR1 relative to that of AtPYR1, which may be a result of an altered orientation of the conserved residues on the contacting interface (Fig. 3D). Nevertheless, the residues within the interface are conserved between the FePYR1 and AtPYR1 (Fig. 3E). These observations suggest that FePYR1 employs a conserved yet somewhat distinct mechanism in regulating downstream PP2C proteins.

To determine the role of the key residues in ABA-dependent interaction of FePYR1 with the co-receptor AtHAB1, mutants of FePYR1 with mutated amino acids in the binding pocket, the gate and the latch were generated and FePYR1-ABA-AtHAB1 interaction was analyzed by AlphaScreen assay (Fig. 3F). Under a saturated concentration of ABA (100 µM), all mutants but the E160A substitution in the pocket and the E131A substitution in the latch exhibited reduced or abolished ABA-dependent interaction with AtHAB1. However, under lower concentrations (10 µM and 2 µM) of ABA, even E160A and E131A displayed reduced ABA-dependent interaction with AtHAB1, but still showed ABA dependence in interaction with AtHAB1 (Fig. 3G). These results revealed critical amino acids in the three well conserved regions of the ABA receptor that are required for ABA binding and ABA-dependent interaction with downstream PP2Cs.

### Engineered FePYR1 receptors with improved ABA binding affinity

**Figure 4:**
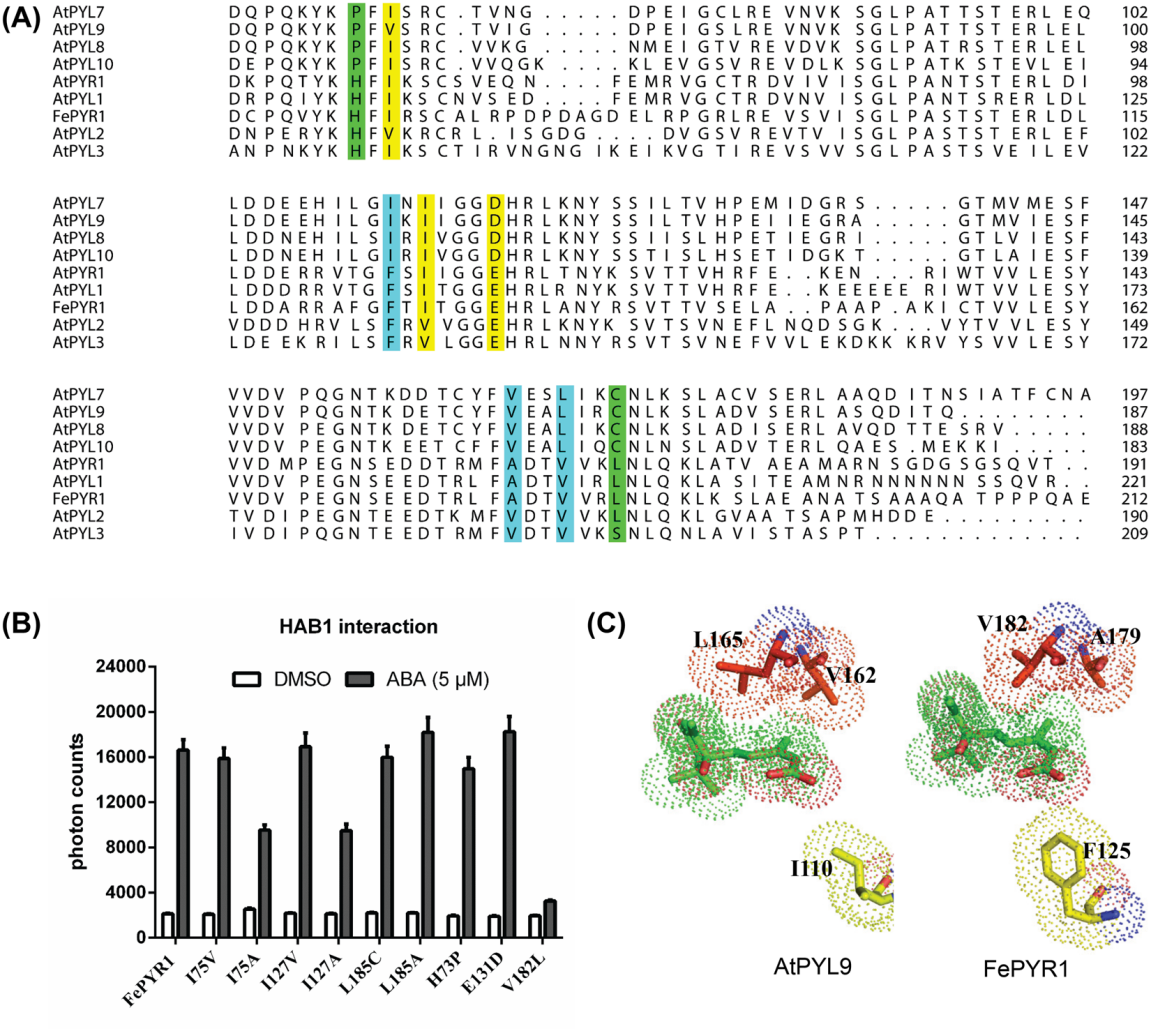
Comparison of FePYR1 with the members of subfamily-I and -III in Arabidopsis. (**A**) Sequence alignment of FePYR1 with subfamily-I and subfamily-III members in *Arabidopsis thaliana*. Variant amino acids in the important regions are highlighted with different colors. (**B**) Interaction of mutants of FePYR1 with indicated amino acid substitutions with AtHAB1 in the presence of 5 μM ABA assessed by Alpha-Screen assay. (**C**) A comparison view of the non-polar triplet residues from AtPYL9 and FePYR1 in contact with ABA. The ABA molecules (green) and triplet residues are shown as stick models with their van der Waals surfaces (dots).

Although FePYR1 has the highest sequence identity with AtPYR1, FePYR1 displayed a monomeric form in solution which is different from the dimeric AtPYR1 (Fig. 2B). Interestingly, the binding affinity with ABA and the capacity of protein phosphatase inhibition are comparable between these two ABA receptors (Fig. S3A and S3B), which suggests that dimerization of the receptor is not a determining factor for ABA binding and signaling. In *Arabidopsis*, the 14 ABA receptor members are classified into three subfamilies^12^ (Fig. S1A), and the monomeric ABA receptors AtPYL9^7^ and AtPYL8^22^ in the subfamily-I have been shown to have ABA binding affinity that are 50- and 90-fold, respectively, stronger than the subfamily-III members AtPYR1 and AtPYL2^7,9^. Thus, we analyzed the divergent amino acid residues in the important regions of FePYR1 and AtPYL9 and attempted to engineer FePYR1 by substituting these residues with the corresponding residues in AtPYL9. Sequence analysis identified several amino acids in the important regions of FePYR1 that are different from those in AtPYL9, including H73, I75, I127, E131, V182 and L185 (Fig. 4A). Except for the change of V182L, all other amino acid substitutions in FePYR1 with the corresponding residues in AtPYL9 did not affect the ABA-dependent inhibition of AtHAB1 activity (Fig. 4B). Comparison of the AtPYL9 (PDB code:3oqu) structure^23^ with the structure of FePYR1 revealed that the three functionally related residues, Ile110, Val162, and Leu165, inside the ABA-binding cavity of AtPYL9 are replaced by Phe125, Ala179, and Val182 in FePYR1 (Fig. 4C). Based on the structural comparison, we deduced that the substitution of V182L is likely to result in clash of ABA molecule in the binding pocket because of the Leu residue possessing stronger steric constraint than the Val residue. In addition, the three corresponding residues in AtPYL9 can fine-tune the position and orientation of the ABA molecule to form direct bifurcated hydrogen bonds to Asn169 in addition to Lys63 (Fig. S4B). In sharp contrast to AtPYL9, these three residues in FePYR1 prevents the ABA carboxyl group from being oriented toward Asn186 to form direct hydrogen bonds (Fig. S4A). Thus, a triple mutant (V182L, A179V, F125I) of FePYR1 was generated to test whether substitution of all these three residues in FePYR1 by the corresponding residues in AtPYL9 could increase ABA-binding affinity. Furthermore, detailed structural analysis of FePYR1 suggested that the Leu185 located in the intermolecular interface to cause hydrophobic interaction (Fig. S4C) is possibly responsible for potential oligomerization in solution. The Leu185 may also affect the His73 orientation, which results in the formation of a hydrogen bond between the His73 main chain and the Asn186 side chain and prevents hydrogen bond formation between the Asn186 side chain and ABA (Fig. S4A). Therefore, a double mutant (L185C, H73P) was also designed to test its ABA-binding affinity. In the members of subfamily-III of ABA receptors, a conserved Glu residue (E131 in FePYR1), which is in the latch loop and prevents closure of the SGLPA gate in the apo-structure, would expend extra energy to flip ~150 degrees for comfortable ABA entrance into the binding pocket. A conserved smaller residue Asp is substituted at this position in the subfamily-I members. Similarly, another two Ile residues, I75 and I127, residing in the ABA-binding pocket (Fig. 3B) may also affect the stereo-selectivity and binding affinity of ABA. Val is a non-polar aliphatic amino acid resembling Ile but smaller in size, and the mutations of I75V and I127V were expected to benefit the entrance of ABA into the binding pocket. Considering the efficiency of the conformational change during the ABA-binding process, a triple mutant (I75V, I127V, E131D) was also generated and tested for its ABA-binding affinity.

**Figure 5:**
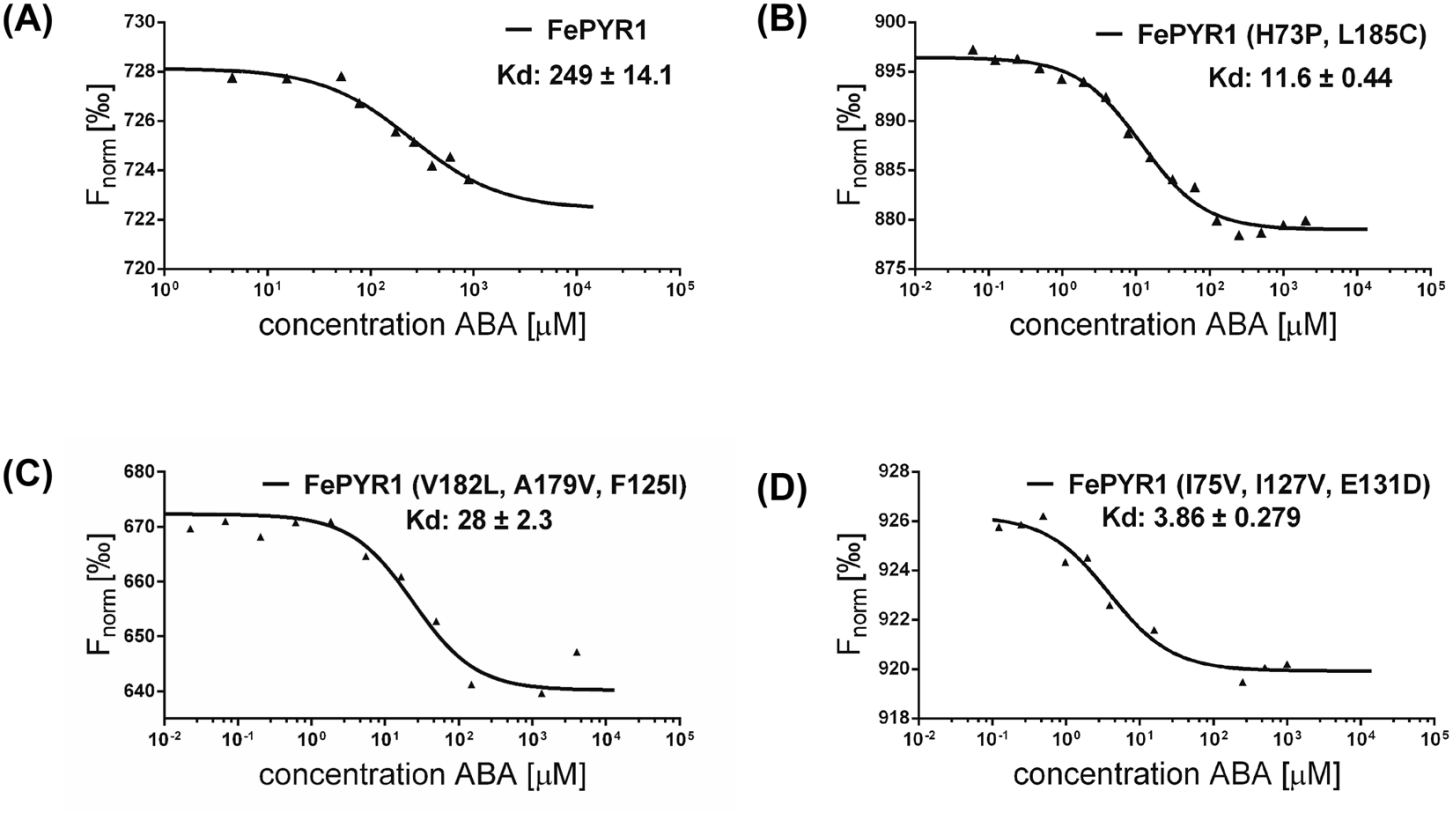
ABA binding affinity of FePYR1 and its AtPYL9-like mutants. (**A**) ABA binding assay of the wild-type FePYR1. 5 μM fluorescently labeled FePYR1 protein was titrated from about 2000 μM to about 4 μM of ABA diluted at 2:3 gradient concentration. The Kd is fitted to 249 ± 14.1 μM. (**B**) ABA binding assay of the double mutant FePYR1-H73P/L185C. 5 μM of the mutant protein was fluorescently labeled and titrated from about 2000 μM of ABA diluted at 1:2 gradient concentration. The Kd is fitted to 11.6 ± 0.44 μM. (**C**,**D**) ABA binding assay of the triple mutants FePYR1-V182L/A179V/F125I and FePYR1-I75V/I127V/E131D. 5 μM fluorescently labeled mutant protein was titrated from about 2000 μM of ABA diluted at 1:3 (**C**) or 1:2 (**D**) gradient concentration, respectively. The Kd is fitted to 28 ± 2.3 μM and 3.86 ± 0.28 μM respectively. The analysis of thermophoresis is plotted by normalized fluorescence [‰] and random fluorescence intensity is ruled out when calculating the fit curve.

ABA-binding affinity of FePYR1 and its mutants was assessed using MST assay. Although FePYR1 resembles AtPYL9 as a monomeric form in solution, FePYR1 exhibited a low binding affinity to ABA with a Kd ~249 μM (Fig. 5A). Surprisingly, the mutations of L185C/H73P, which are located at the intermolecular interface and may function in dimer formation, converted FePYR1 to a 20-fold higher affinity state with a Kd value of 11.6 μM (Fig. 5B). The triple mutations (V182L, A179V, F125I) in the ABA-binding pocket also enhanced the ABA affinity about 9-fold with a Kd value of ~28 μM (Fig. 5C). The engineered mutations (I75V, I127V, E131D) resulted in highest ABA-binding affinity with a Kd of ~3.86 μM (Fig. 5D). All these three structure-guided engineered forms of FePYR1 can be considered as AtPYL9-like receptors and displayed superior ABA-binding affinity than its wild type form. The physiological significance of improved ABA-binding affinity of the engineered FePYR1 was further supported by the measurements of ABA contents in both *Arabidopsis* and turfgrass (Fig. S5). Under well-watered conditions, the ABA contents were ~7.63 ng/g FW (~31 nM) in the *Arabidopsis* leaves and ~2.93 ng/g FW (~12 nM) in the turfgrass leaves. When soil water content reduced to 25%, the ABA contents increased to ~22.47 ng/g FW (~0.10 µM) in *Arabidopsis* (~89% water content in leaves) and ~251.97 ng/g FW (~1.28 µM) in turfgrass (~74% water content in leaves). The ABA contents further increased to ~108.93 ng/g FW (~0.52 µM) in *Arabidopsis* (~80% water content in leaves) and 335.60 ng/g FW (~2.31 µM) in turfgrass (~55% water content in leaves) when soil water content reduced to 10%. These results indicate that the ABA levels accumulated to a micromolar range in the turfgrass *Festuca elata* after moderate to severe drought stress, within which the engineered FePYR1, but not the wild type FePYR1, would operate to mediate ABA signaling. Thus, structure-guided design could be used to create more efficient ABA receptors, which could be used for improving abiotic stress resistance in turfgrass and other crops.

### FePYR1 could be used to improve drought resistance in plants

**Figure 6:**
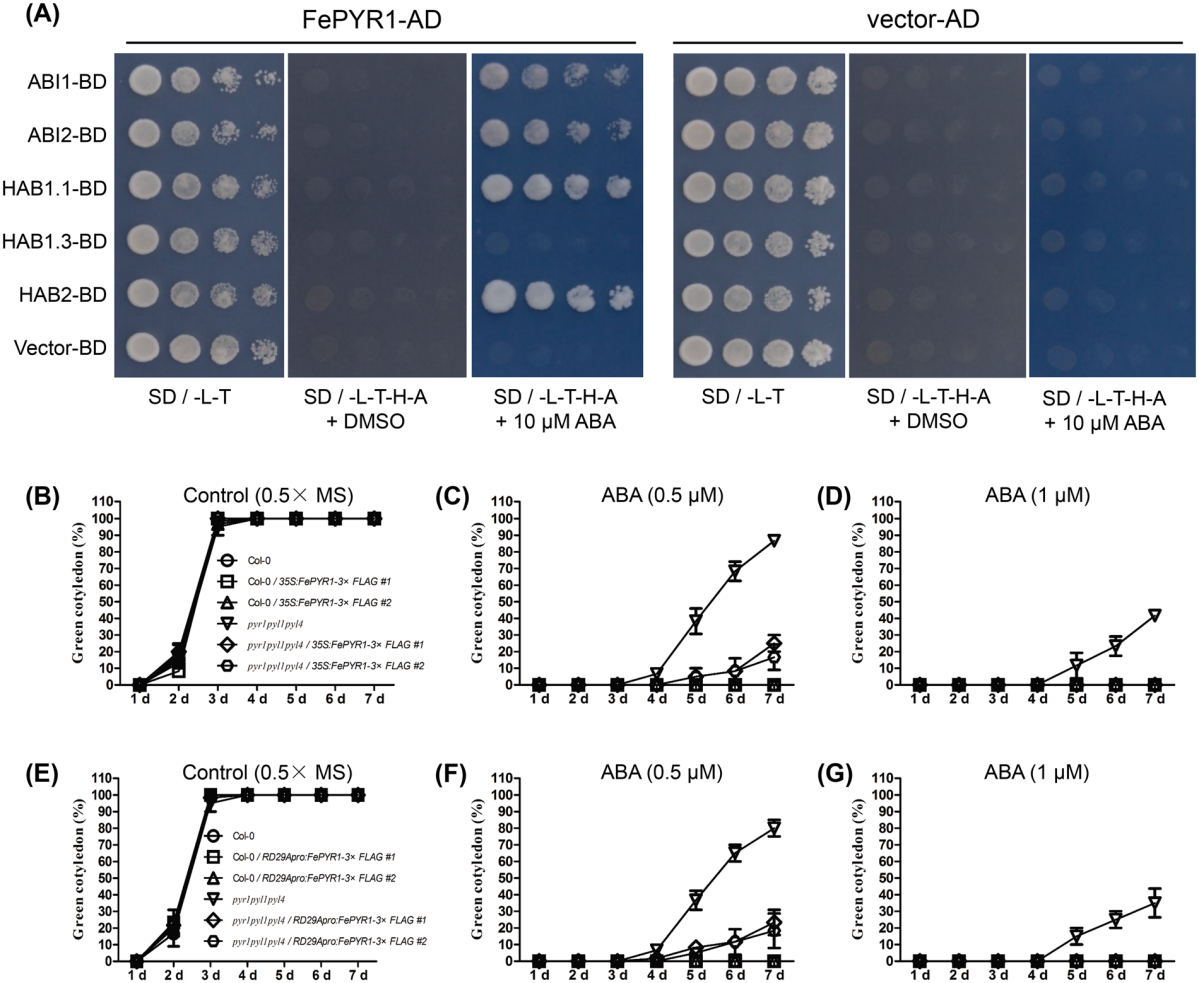
FePYR1 interacts with the Arabidopsis PP2Cs and rescues the ABA insensitive phenotype of *pyr1pyl1pyl4* mutant. (**A**) Yeast-two-hybrid assay to determine interaction of FePYR1with the indicated downstream PP2C proteins. (**B**–**G**) Seed germination assay of FePYR1 transgenic plants driven by the 35 S promoter (**B**–**D**) and FePYR1 transgenic plants driven by the inducible *RD29A* promoter (**E**–**G**) in 0.5 × MS media supplemented with 0 μM (**B**,**E**), 0.5 μM (**C**,**F**) and 1 μM (**D**,**G**) ABA. Wild type (Col-0) and *pyrl1pyl1pyl4* mutant plants were used as controls. The germination rate of all genotypes were counted at the 7th day after sowing. Error bars are means ± SD of biological triplicates. Each treatment contained at least 50 seeds.

Yeast two-hybrid assay revealed that FePYR1 could interact with the *Arabidopsis* clade A PP2C enzymes ABI1, ABI2, HAB2 and the functional HAB1 variant HAB1.1, but not the non-functional splicing variant HAB1.3, in the presence of 10 μM ABA, while without ABA, FePYR1 could not interact with these PP2C proteins (Fig. 6A). This indicates that FePYR1 interacts with the *Arabidopsis* PP2Cs in an ABA-dependent manner. The results also suggest that FePYR1 could be sufficient to activate ABA-signaling pathways and enhance stress resistance in *Arabidopsis*.

We thus generated transgenic plants in *Arabidopsis* wild type and *pyrl1pyl1pyl4* triple mutant backgrounds that express FePYR1 under the control of the constitutive 35 S promoter or the stress-inducible *RD29A* promoter. Constitutive and induced expression of FePYR1 in different transgenic plants were verified by both quantitative RT-PCR and Western blotting (Fig. S6). The biological activity of FePYR1 in *Arabidopsis* was assessed by a classical seed germination assay. As shown in Fig. 6B–G, in the wild type Col-0 background, all FePYR1 transgenic lines exhibited an ABA-sensitive phenotype similar with wild type plants. The *pyrl1pyl1pyl4* triple mutant plants showed an ABA-insensitive phenotype as expected, and expression of FePYR1 partially rescued the ABA-insensitive phenotype of *pyr1pyl1pyl4* (Fig. 6C,F and Fig. S7). These results indicate that FePYR1 is biologically active and could function as an ABA receptor in planta.

**Figure 7:**
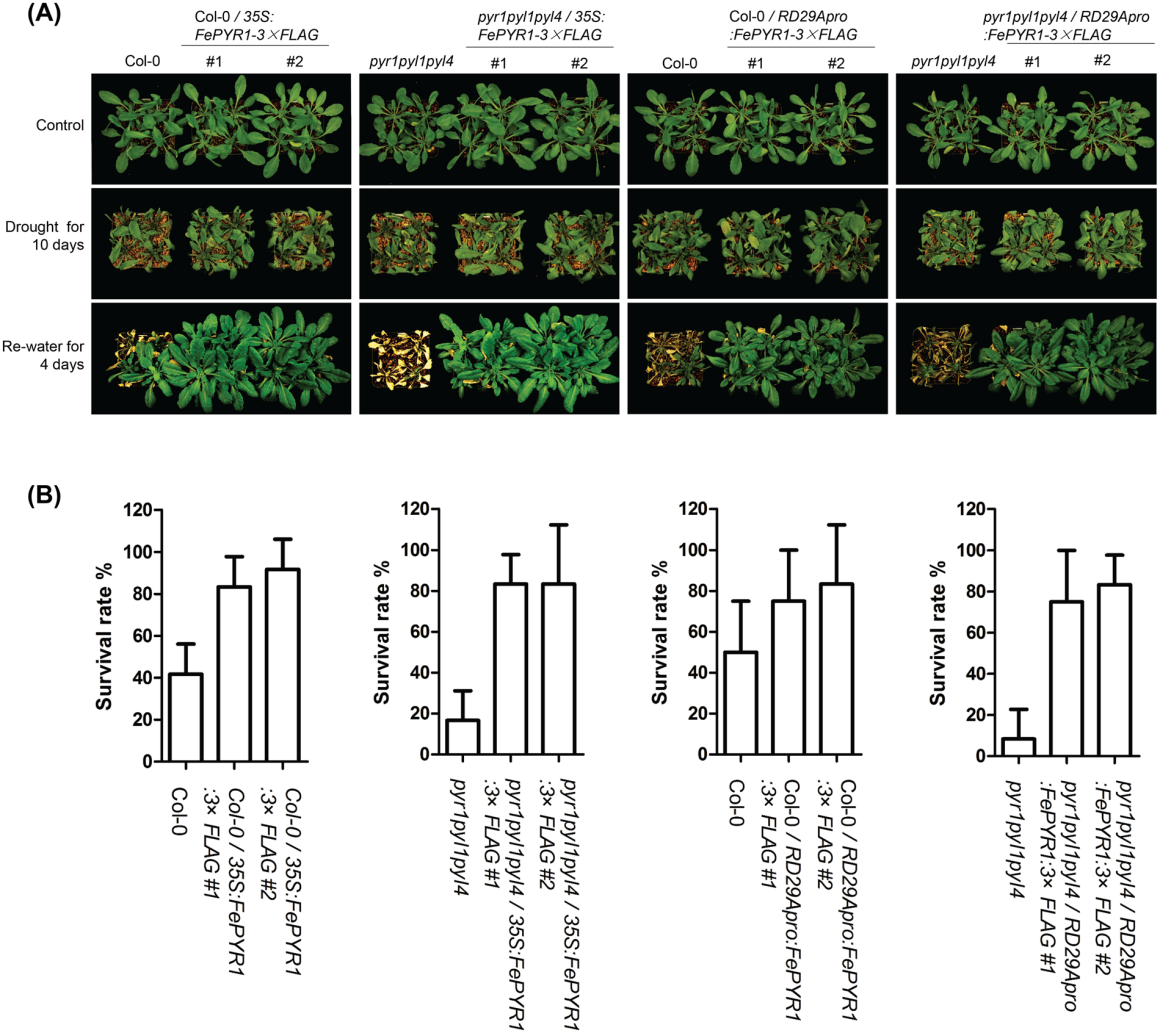
Expression of FePYR1 improves drought resistance in Arabidopsis. (**A**) Drought treatment assay of 21-day-old FePYR1 transgenic lines, wild type (Col-0) and *pyr1pyl1pyl4* mutant plants grown under short-day conditions, water withdrawal for 10 days and re-watered for 4 days before being photographed. (**B**) Quantitative analysis of survival rate of the genotypes indicated in (**A**). Error bars indicate ±SD of triplicate assays. Each treatment contained 16 plants.

A critical role of ABA-receptor mediated signaling is to adjust the physiological status of plants in response to water deficit^24,25^.Thus, the transgenic lines were further evaluated to determine whether expression of FePYR1 could improve resistance to drought conditions. As shown in Fig. 7, after a 10-day water deprivation and then re-watered for 4 days, all transgenic lines displayed significantly increased survival rates when compared with the Col-0 wild type and *pyrl1pyl1pyl4* triple mutant. These results showed that FePYR1 can be utilized to improve drought resistance in plants.

## Discussion

During evolutionary transition of plants from aquatic to terrestrial environments, a core ABA-signaling pathway emerged, which is thought to be a milestone for plants to cope with drought conditions^18^. ABA signaling is mediated by the ABA receptors as the first signaling event, thus the binding affinity of ABA receptors with ABA and selection of the downstream interacting PP2Cs are critical factors determining the intensity of molecular and physiology response of the plants to drought stress. Therefore, manipulation of ABA receptors has been considered as a biotechnological tool for improving drought resistance in plants^26^. For example, structure-guided engineering of ABA receptor proteins and structure based rational design of chemicals with superior binding affinity with ABA receptors have been reported to have great potential in agriculture for stress resistance^16,26,27^. However, these approaches require the structural information of ABA receptors, and crystal structures of ABA receptors are still very limited. Moreover, plants have diversified ABA receptors in both primary sequences and tertiary structures. Therefore, elucidating the crystal structures of ABA receptors in agronomically and economically important plant species and finding the structural differences responsible for distinct ABA binding affinities would help in structure-guided design of both superior ABA receptors and small molecules with higher potency and stability than ABA. In this study, we characterized putative ABA receptors in turfgrass in anticipation of manipulating the ABA-signaling pathway thus improving drought resistance in this important group of ornamental plants. Sequence analysis and biochemical study identified several ABA receptors from turfgrass, and one of these receptors, FePYR1, was confirmed as an authentic ABA receptor. FePYR1 exhibited a classical α-β-α2-β6-α arrangement (Fig. 1C), a topological hallmark of ABA receptors in the model plant *Arabidopsis* ^21^. By analyzing the crystal structure of FePYR1-ABA complex, we found that the ABA receptor FePYR1 also employs the so-called gate-latch-lock mechanism that operates in *Arabidopsis* for receptor-ABA binding (Fig. 3), which is consistent with the ubiquitous role of ABA in the plant kingdom. However, FePYR1 was also found to be somewhat distinct from the *Arabidopsis* AtPYR1. Unlike AtPYR1 which functions as a dimer^10^, FePYR1 is a monomer in solution (Fig. 2B). In addition, although some of the residues located in the interface with the downstream PP2C proteins are conserved, the orientation and capacity to interact with PP2C proteins differ between FePYR1 and AtPYR1 (Fig. 3D–G). Such structural differences among ABA receptors may result in different ABA-binding affinities and association with different downstream components, which consequently leads to distinct functions of the ABA receptors within and among plant species.

All higher plants possess a family of ABA receptors, whose binding affinities with ABA and downstream interaction partners are diversified. For example, the classical monomeric receptor AtPYL9 has a high ABA-binding affinity with a Kd of 0.66 μM^7^. However, the dimeric receptor AtPYL2 has a low affinity with a Kd of 59 μM^9^. In addition, a study showed that the dimeric receptor AtPYL2 lost nearly all inhibitory ability to AtHAB1 when the AtPYL2 was present at less than a half of AtHAB1 concentration, while the monomeric receptors still function well at low concentrations^28^. Our study revealed that FePYR1 exists as a monomer resembling the *Arabidopsis* subfamily-I members such as AtPYL9, but it has a low ABA-binding affinity (Fig. 5A) similar as the *Arabidopsis* subfamily-III members like AtPYR1. This suggests that oligomerization is not a determining factor for ABA-binding affinity but variations in the critical regions of the receptors may play more important roles in ABA binding and interactions with PP2Cs.

By sequence comparison of FePYR1 and the *Arabidopsis* subfamily I and III members, several amino acid variations in the critical regions of the receptors were identified between FePYR1 and AtPYL9 (Fig. 4A). Whether these variant amino acids play important roles in ABA-binding affinity was determined by substitution of these amino acids in FePYR1 with the corresponding amino acids in AtPYL9. Three mutants that were rationally designed based on the structural comparisons (Fig. 4C, Fig. S4) showed remarkably increased ABA-binding affinity (Fig. 5), further supporting that amino acid substitutions in the critical functional regions of the ABA receptors are responsible for ABA-binding affinities. Moreover, ABA content measurements (Fig. S5) indicated that the engineered FePYR1 receptors should be operational within the physiological levels of ABA in the turfgrass under drought stress conditions. Therefore, our results suggest that changing a few amino acids could create ABA receptors with superior ABA-binding affinity, which are expected to trigger stronger responses to ABA in plants under abiotic stress conditions such as drought. Although the wild type FePYR1 has a low ABA-binding affinity, expression of FePYR1 significantly improved drought resistance in *Arabidopsis* (Fig. 7). In the future, it would be interesting to test whether the mutants of FePYR1 with increased ABA-binding affinity could be superior to wild type FePYR1 in conferring drought resistance in plants. Overall, our study provided detailed structural information about the turfgrass ABA receptor FePYR1 and confirmed that engineering of the receptor by changing a few amino acid residues can significantly enhance its ABA-binding affinity. These findings could further assist future structure-guided design of superior ABA receptors as well as small molecules that are individualized for specific plant species.

## Materials and Methods

### DNA constructs and reagents

Based on the transcriptome sequences of *Festuca elata* provided by Dr. Zhulong Chan (Huazhong Agricultural University) and Zoysia japonica^29^, the cDNAs encoding FePYLs (FePYR1, FePYL1-FePYL5) and ZjPYLs (ZjPYL1-ZjPYL3) were synthesized by Genewiz and were cloned into the pSumo expression vector (LifeSensors) with a H6-SUMO tandem fusion tag followed by an ULP1 protease cleavage site at the N-terminus. For AlphaScreen and phosphatase activity assays, AtPYR1 (residues 9-182) fused with H6-SUMO and PP2C protein AtHAB1 (residues 172–511) with a Biotin-MBP tag are used as the positive control^16^. For yeast two-hybrid assay, the *FePYR1* CDS was cloned into the pGBDT7 vector (Clontech) with a GAL4 DNA binding domain at the N-terminus. The *ABI1*, *ABI2*, *HAB1* isoforms, and *HAB2* CDS were cloned into the pGADT7 vector (Clontech) with a GAL4 activation domain at the N-terminus. To generate transgenic lines expressing FePYR1-3 × Flag in *Arabidopsis*, the *FePYR1* cDNA driven by the 35 S promoter or *RD29A* promoter was cloned into pCAMBIA1305 vector containing a 3 × Flag tag, and then transferred into *Agrobacterium tumefaciens* strain GV3101 for *Arabidopsis* transformation using standard floral dip method^30^.

### Protein expression and purification

All 6 FePYLs, 3 ZjPYLs and AtPYR1 were expressed as H6-SUMO fusion proteins. For crystallization, the expression plasmids were transformed into *E. coli* BL21 (DE3) cells and transformants were grown in 2 L of LB media at 37 °C. At the cell density of OD_600_ of ~1.0, a final concentration of 0.1 mM IPTG was added to induce protein expression at 16 °C overnight. Cells were then harvested, re-suspended in 50 mL Buffer A (20 mM Tris (pH 8.0), 200 mM NaCl, 10% glycerol) and lysed with a French press at 800 Pa. The lysate was centrifuged for 30 min at 16,000 rpm and the supernatant was loaded onto a 50 mL Ni-chelating HP Sepharose column (GE Healthcare). The protein was eluted using step elution in Buffer A with 250 mM imidazole and cleaved with ULP1 protease at a protease/protein ratio of 1:1000 during dialysis against Buffer A at 4 °C overnight. The cleaved H6-SUMO tag was removed by passing through a 5 mL Ni-chelating HP Sepharose column (GE Healthcare), and the protein in the flow-through was further purified by gel filtration chromatography using a 320 mL HiLoad 26/60 Superdex 200 column (GE Healthcare) and elution solution containing 20 mM Tris (PH 8.0) and 200 mM ammonium acetate. The protein eluted from the gel filtration column was collected at a volume corresponding to the size at a purity >95% as determined by SDS-PAGE (Fig. S2).

For AlphaScreen and phosphatase activity assay, H6-SUMO-tagged FePYLs, ZjPYLs, AtPYR1 and FePYR1 mutant proteins, as well as the biotin-MBP-tagged AtHAB1, were expressed in *E. coli* in 100 mL of LB medium by induction with 0.1 mM IPTG for 16 h at 16 °C. In addition, 40 µM biotin also was added to the culture to induce the biotin-MBP-tagged AtHAB1. Small scale purification of these proteins was performed as previously described^8^.

### AlphaScreen binding assay

Interactions between H6-SUMO-tagged FePYLs, ZjPYLs and mutants of FePYR1 with biotinylated AtHAB1 (PP2C) were assessed by luminescence-based AlphaScreen technology using a hexahistidine detection kit (Perkin Elmer, Cat#67660619 M)^8,31,32^. Unless otherwise noted, 100 nM biotinylated AtHAB1 proteins were attached to streptavidin-coated donor beads and 100 nM H6-SUMO-tagged PYL proteins were attached to nickel-chelated acceptor beads with or without the indicated amounts of ( + )-ABA. When excited by a laser beam of 680 nm, the donor beam emits singlet oxygen that activates thioxene derivatives in the acceptor beads to release photons of 520–620 nm as the binding signal, which could be detected by the Envision Multilabel Reader (Perkin Elmer, Cat#2014-0010). For dose-response assay, the concentrations of ( + )-ABA ranging from 2 nM to 300 μM were used. For competition assay, the untagged proteins at concentrations of 0 μM to 50 μM were added in addition to the tagged proteins. Each data point was an average of triplicate measurements. The IC_50_ values were determined by curve fitting based on a competitive inhibitor model using GraphPad Prism. The assay conditions for IC_50_ were same as the AlphaScreen binding assay described above.

### Crystallization, data processing and structure determination

Purified putative ABA receptor proteins FePYL1, FePYR1 and ZjPYL2 were concentrated by ultrafiltration to about 10 mg/mL (determined by Bradford assay) prior to crystallization trials. To prepare PYL-ABA and PYL-ABA-HAB1 complex, ABA was added to PYL proteins with 5:1 molar ratio and to the mixture of PYL and HAB1 proteins with 5:1:1 molar ratio. After overnight incubation, initial screening was carried out by using the Hampton screening kit (Hampton Research, Aliso Viejo, CA 92656-3317 USA). Many different conditions yielded protein crystals with the sitting drop method at 20 °C, but only FePYR1-ABA complex formed crystals suitable for diffraction and other crystals of PYL-apo proteins, PYL-ABA and PYL-ABA-HAB1 were all needle shaped and too small to diffract. The FePYR1-ABA complex crystals were initially grown in a condition containing 0.1 M BIS-TRIS (pH 6.5), 1.8 M ammonium sulfate, 2% polyethylene glycol monomethyl ether 550. The crystal grown conditions for FePYR1-ABA were further optimized using hanging drop method with 1.0 μL of purified FePYR1-ABA protein and 1.0 μL of well solution at 20 °C. The optimal conditions for FePYR1-ABA crystals were determined as 0.1 M BIS-TRIS propane pH 7.0, 1.5 M ammonium sulfate. All crystals appeared within 4 days and were then flash frozen in liquid nitrogen. All diffraction data were collected at 100 K using an X-ray beam at BL17U beamline at the Shanghai Synchrotron Radiation Facilities. The diffraction data were reduced and scaled using MOSFLM^33^ and SCALA^34^. The structure was then solved by molecular replacement using Phaser^35^ with the structure model of *Arabidopsis* PYL2 (PDB code: 3KB0) as search model. COOT^36^ was used to manually rebuild the structure model, and the structure was further refined using Phenix software package^37^. The program Voidoo^38^ was used to determine the size of the ABA-binding pocket. All the structure figures presented in this paper were prepared by using PyMOL (DeLano Scientific). A summarized statistics of data collection and structure determination are shown in Table S1.

### AtHAB1 phosphatase inhibition assay

The Biotin-MBP-HAB1 protein at a concentration of 100 nM and H6-SUMO-tagged PYR/PYLs at a concentration of 500 nM were pre-incubated in 50 mM imidazole pH 7.2, 5 mM MgCl_2_, 0.1% β-mercaptoethanol, 0.5 µg/mL BSA and different concentrations of ABA for 20 min at room temperature, as previously described^8^. The synthesized phosphopeptide (HSQPKpSTVGTP), corresponding to the amino acids from 170–180 of SnRK2.6 with Ser175 phosphorylated, was added as a substrate of AtHAB1 phosphatase. Thephosphopeptide at a concentration of 100 μM was then added to initiate the reaction, and phosphate released from the phosphopeptide was determined by colorimetric assay (BioVision) after 35 min reaction.

### Microscale thermophoresis (MST) assay

MST assay was performed with the Monolith NT™ Protein Labeling Kits and the Monolith NT115 series instrument (NanoTemper Technologies GmbH, 81369 München, Germany) as previously described^39,40^. For protein labeling, two to twenty micromolar protein in the Labeling Buffer was mixed with 2- to 3-fold concentration of the protein labeling fluorescent dye, and then incubated for 30 min at room temperature in dark. The labeling reaction was then adjusted to 500 µL and loaded to the Column B. The flow through was discarded and the labelled proteins in column were eluted with 600 µL of storage/analysis buffer (20 mM HEPES pH 7.5, 200 mM NaCl) and collected as aliquots. Before the ABA-binding affinity assay, labeling efficiency was evaluated using the Monolith.NT115 series instrument. For ABA-binding affinity assay, ten to sixteen different concentrations of ABA by series dilutions were used. ABA was mixed with the labeled protein samples, incubated for 5 min and the fluorescent signal was measured by using the Monolith NT115 series instrument with standard treated capillaries (K002). The signal intensity was interpreted by the MST analysis software.

### Measurement of ABA content in plants

4-week-old *Arabidopsis thaliana* (Col-0) and 1-week-old *Festuca elata* grown under 10-h light/14-h dark photoperiod were subjected to drought stress treatments until the soil moisture reached to 50%, 25% and 10% respectively. 100 mg of leaves from well-watered (100% soil moisture) and drought-stressed plants were harvested for ABA measurements. ABA were extracted from leaves with homogenization buffer (70% methanol, 0.1% formic acid, 4 ng/mL ABA-d6) and measured by UPLC-Triple TOF 5600+ system (Sciex, Concord, Canada).

### Yeast two-hybrid assay

Yeast two-hybrid assay was carried out as described previously^7,8^. The open reading frames of *FePYR1* and five PP2C genes were cloned into pGBDT7 and pGADT7 vector (Clontech) respectively. The fusion constructs were verified by sequencing, and corresponding bait and prey constructs were co-transformed into the yeast strain AH109 (Clontech). After incubation at 30 °C on (-L/-T) SD agar plates for two days, well-grown clones were selected and cultured in (-L/-T) SD liquid media overnight. The culture was then diluted to OD_600_ ~0.3, which was further diluted to 1:10 and 1:100 dilutions. One microliter of each dilution was spotted on SD agar plates lacking Leu, Trp, His, and Ade (-L/-T/-H/-A), with or without 2 μM ABA. The assay was carried out using three different clones and photograph was taken after incubation at 30 °C for two days.

### Plant materials and growth conditions

*Arabidopsis thaliana* ecotype Columbia-0 (Col-0), *PYR*/*PYL* triple mutant (*pyr1/pyl1*/*pyl4*) and *FePYR1* transgenic plants used in seed germination assay and gene expression analysis were grown on half-strength MS (Murashige and Skoog) solid media containing 1% sucrose in an environment-controlled chamber at 23 °C with a photosynthetically active radiation of 75 µmol/m^2^/s^1^ and a 16-h light/8-h dark photoperiod. The plant materials used in drought stress assays were grown in soil in a growth room under a relatively short photoperiod (8-h light at 23 °C and 16-h dark at 20 °C).

### Genbank accession numbers and FePYR1 PDB code

The sequence data used in this study were deposited in the Genebank database, and Genbank accession numbers are: FePYR1: KY475596; FePYL1: KY475597; FePYL2: KY475598; FePYL3: KY475599; FePYL4: KY475600; FePYL5: KY475601. The FePYR1 structure data was deposited in the Protein data bank, the PDB code is 5UJV.

## Electronic supplementary material

Supplementary Information
