# Supplementary material for: Structure determination and activity manipulation of the turfgrass ABA receptor FePYR1
Source: Sci Rep. 2017 Oct 25;7:14022. doi: 10.1038/s41598-017-14101-9 (PMC5656587; doi:10.1038/s41598-017-14101-9)
Supplement: Supplementary file 1 — Supplementary Information [file 41598_2017_14101_MOESM1_ESM.pdf]

Supplemental information for:

Structure determination and activity manipulation of the turfgrass ABA receptor FePYR1

Zhizhong Ren[1, 2,#], Zhen Wang[1, 2,#], X Edward Zhou[3], Huazhong Shi[4], Yechun Hong[1, 2], Minjie Cao[1], Zhulong Chan[5], Xue Liu[1], H Eric Xu[3, 6, *], Jian-Kang Zhu[1, 7, *]

[1]*Shanghai Center for Plant Stress Biology and Center for Excellence in Molecular Plant Sciences, Chinese Academy of Sciences, Shanghai 200032, China;* [2]*University of Chinese Academy of Sciences (CAS), Shanghai 200032, P.R. China;* [3]*Laboratory of Structural Sciences and Laboratory of Structural Biology and Biochemistry, Van Andel Research Institute, Grand Rapids, MI, USA;* [4]*Department of Chemistry and Biochemistry, Texas Tech University, Lubbock, Texas 79409;* [5]*Key Laboratory of Horticultural Plant Biology, Ministry of Education, College of Horticulture & Forest Sciences, Huazhong Agricultural University, Wuhan 430070, China;* [6]*Key Laboratory of Receptor Research, VARI-SIMM Center, Center for Structure and Function of Drug Targets, Shanghai Institute of Materia Medica, Chinese Academy of Sciences, Shanghai, China;* [7]*Department of Horticulture and Landscape Architecture, Purdue University, West Lafayette, Indiana 47907*

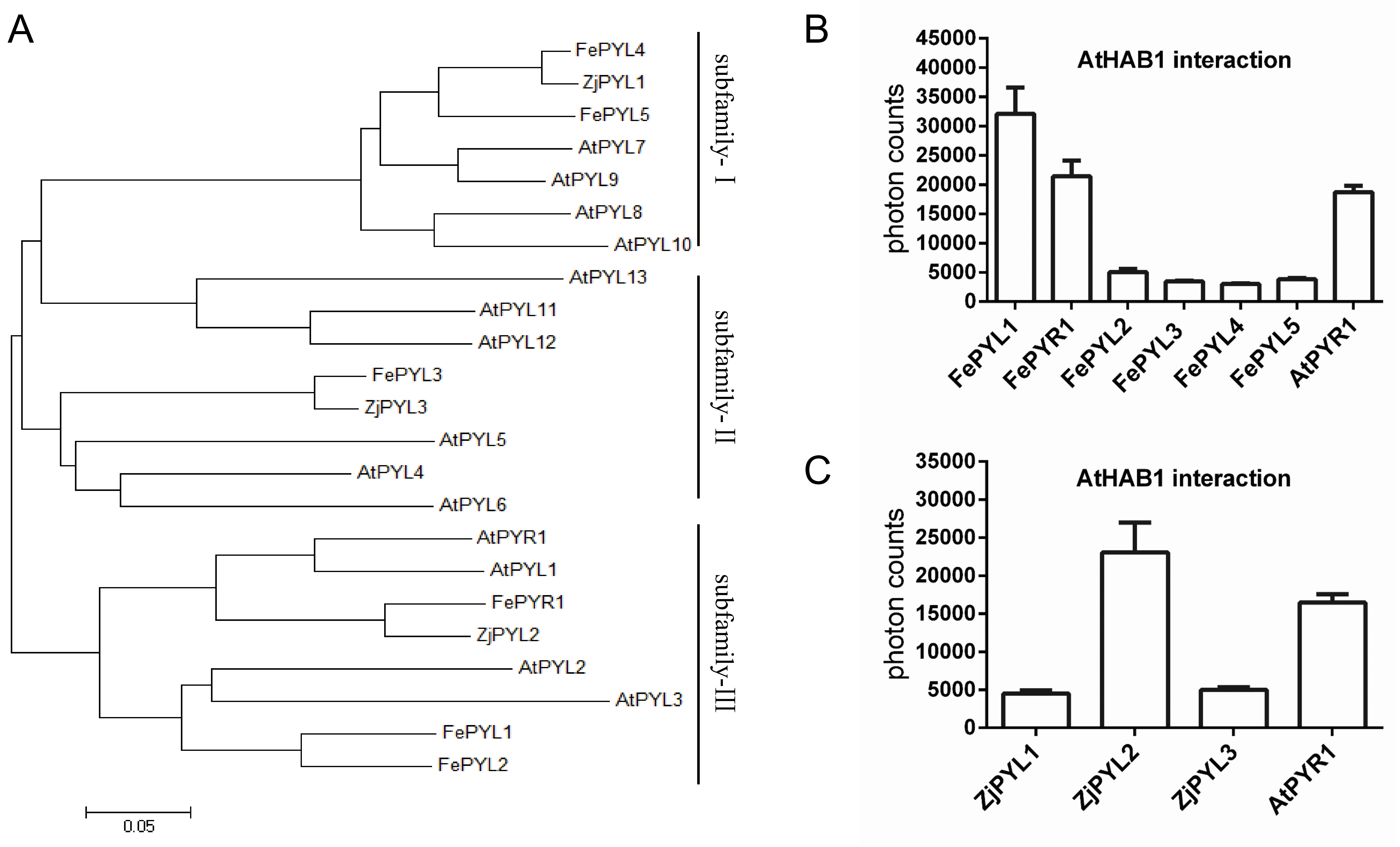

Supplemental Figure 1. Determination of ABA receptors in the two turfgrass species. (A) A phylogeny of multiple sequence alignment of the turfgrass and *Arabidopsis* PYR/PYLs using the neighbor joining method in MEGA5. These proteins were divided into three major subfamilies I, II and III to their phylogenic relationships. (B) Identification of *Festuca elata* ABA receptors by Alpha-Screen assay. Interactions of putative receptor proteins with the *Arabidopsis* AtHAB1 were evaluated by photon intensity (n=3, error bars are means ± SD). (C) Determination of *Zoysia japonica* ABA receptor proteins using Alpha-Screen assay. Interactions between ZjPYR/PYLs candidates and AtHAB1 were analyzed by photon counts (n=3, error bars represent ± SD).

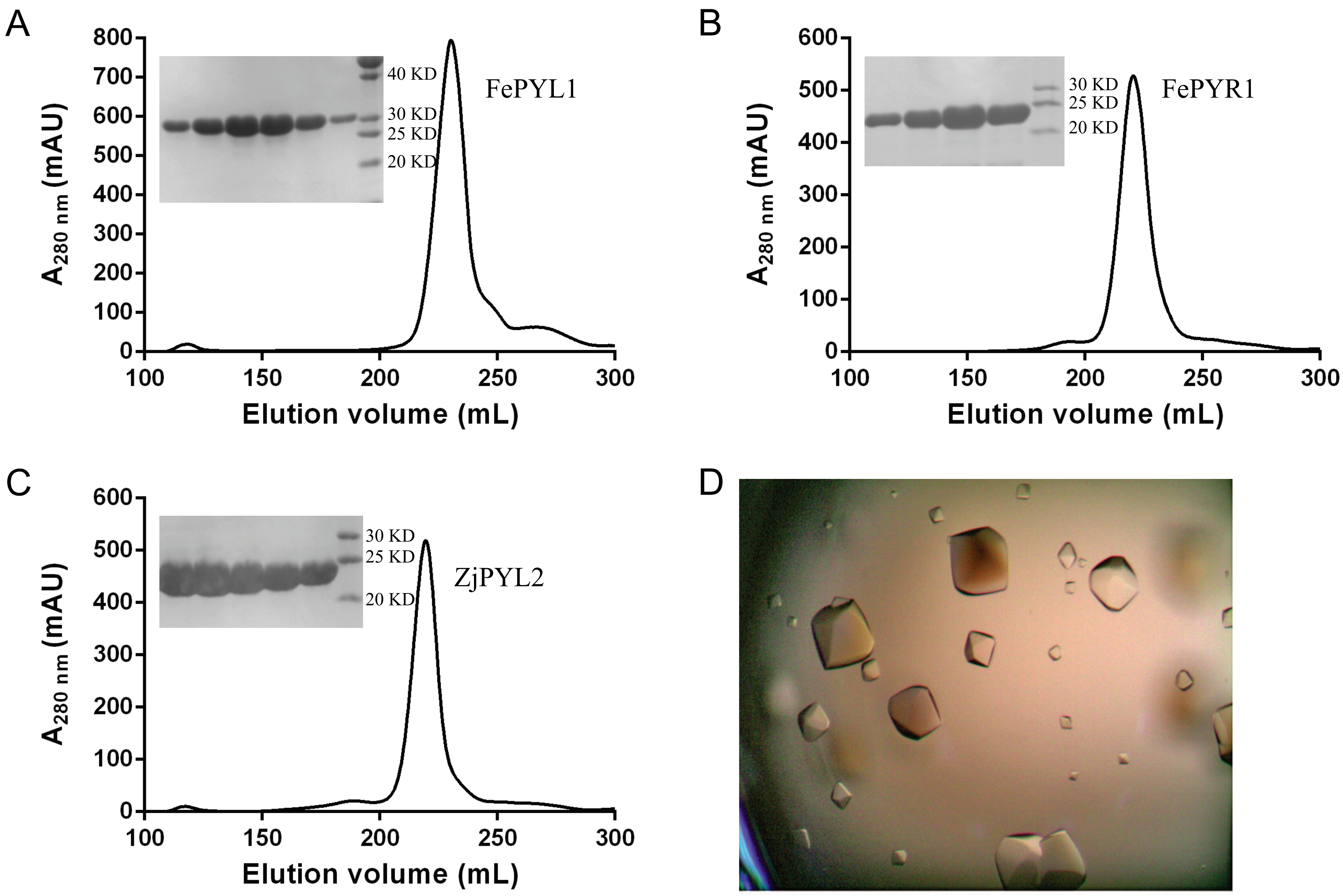

Supplemental Figure 2. Purification and crystallization of the three putative turfgrass ABA receptor proteins.

(A-C) The size-exclusion chromatography (SEC) elution profiles for FePYL1, FePYR1 and ZjPYL2 respectively. The proteins in the fractions of SEC eluents were visualized by Coomassie Brilliant Blue staining. (D) Crystals of FePYR1-ABA complex after 4 days hanging drop optimization in the buffer containing 0.1 M Bis-Tris propane (pH 7.0) and 1.5 M Ammonium sulfate.

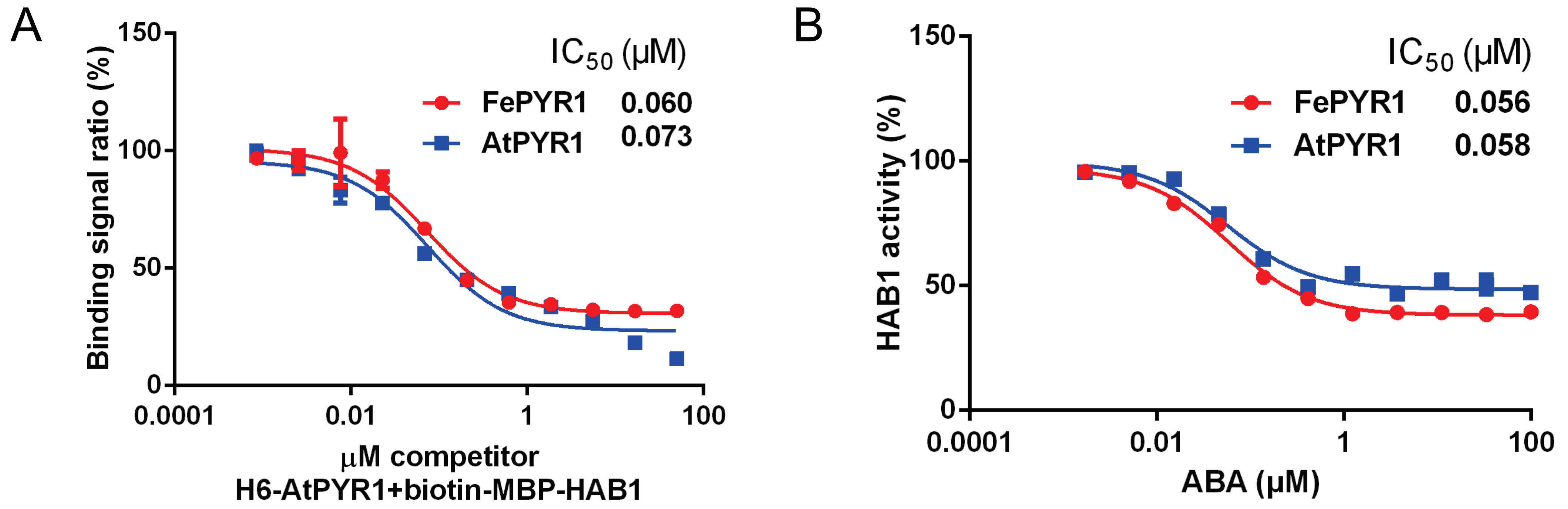

Supplemental Figure 3. Comparison of FePYR1 and AtPYR1 on binding with and inhibition of AtHAB1.

(A) Competition binding of FePYR1 and AtPYR1 analyzed by using luminescent proximity assay. The interaction strength of 100 nM biotinylated AtHAB1 and 100 nM 6×His-fusion AtPYR1 in the presence of 100 μM ABA was used as the initial intensity (100%) in the Alpha-Screen assays described in the Materials and Methods. Addition of unlabeled FePYR1 (red) or AtPYR1 (blue) at the indicated concentrations competed with 6×His-tagged PYR1 for binding to biotinylated AtHAB1 (n=3, error bars represent ± SD). (B) Comparison of inhibition of AtHAB1 phosphatase activity by FePYR1 and AtPYR1. 100 nM biotinylated AtHAB1 and 500 nM H6-SUMO tagged FePYR1 (red) or AtPYR1 (blue) were incubated at indicated concentrations of ABA. AtHAB1 phosphatase activity was determined by colorimetric assay (BioVision) (n=3, error bars mean ± SD).

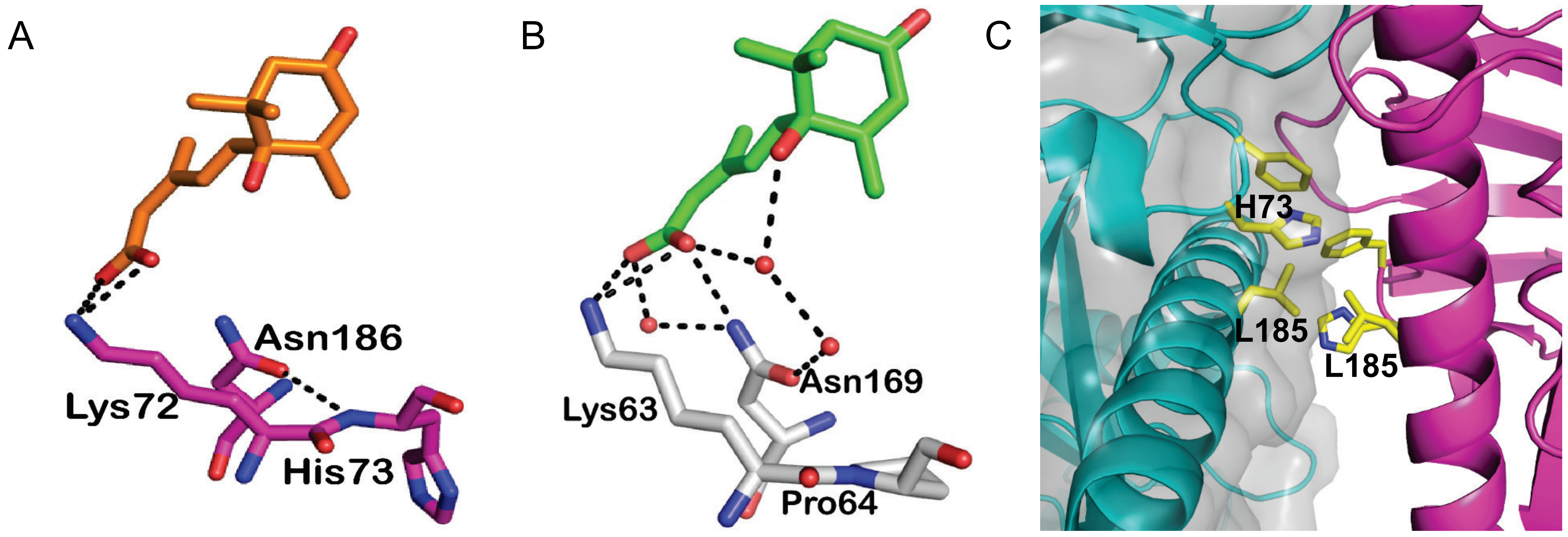

Supplemental Figure 4. Detailed conformational views of ABA in the aligned structures of ABA-FePYR1 and ABA-AtPYL9.

(A, B) The side view of ABA bound to FePYR1 (A) and AtPYL9 (B). The bound ABA molecule (aurantia) in FePYR1 forms hydrogen bonds with Lys72 and has no other direct hydrogen bonds with amino acids at the bottom of FePYR1 pocket. An intra-molecule hydrogen bond is formed between the Asn186 and His73. ABA molecule (green) makes intimate contacts with AtPYL9 by forming direct hydrogen bonds with Asn169 and Lys63. Direct hydrogen bonds between ABA molecule and proteins were shown with broken lines, water molecules were shown as red balls. (C) The detailed structure of FePYR1 interface formed by α3 helix between two adjacent molecules. The backbone of two adjacent FePYR1 molecules were shown in cyan with semitransparent molecular surface and magenta respectively. The relevant residues were shown in yellow.

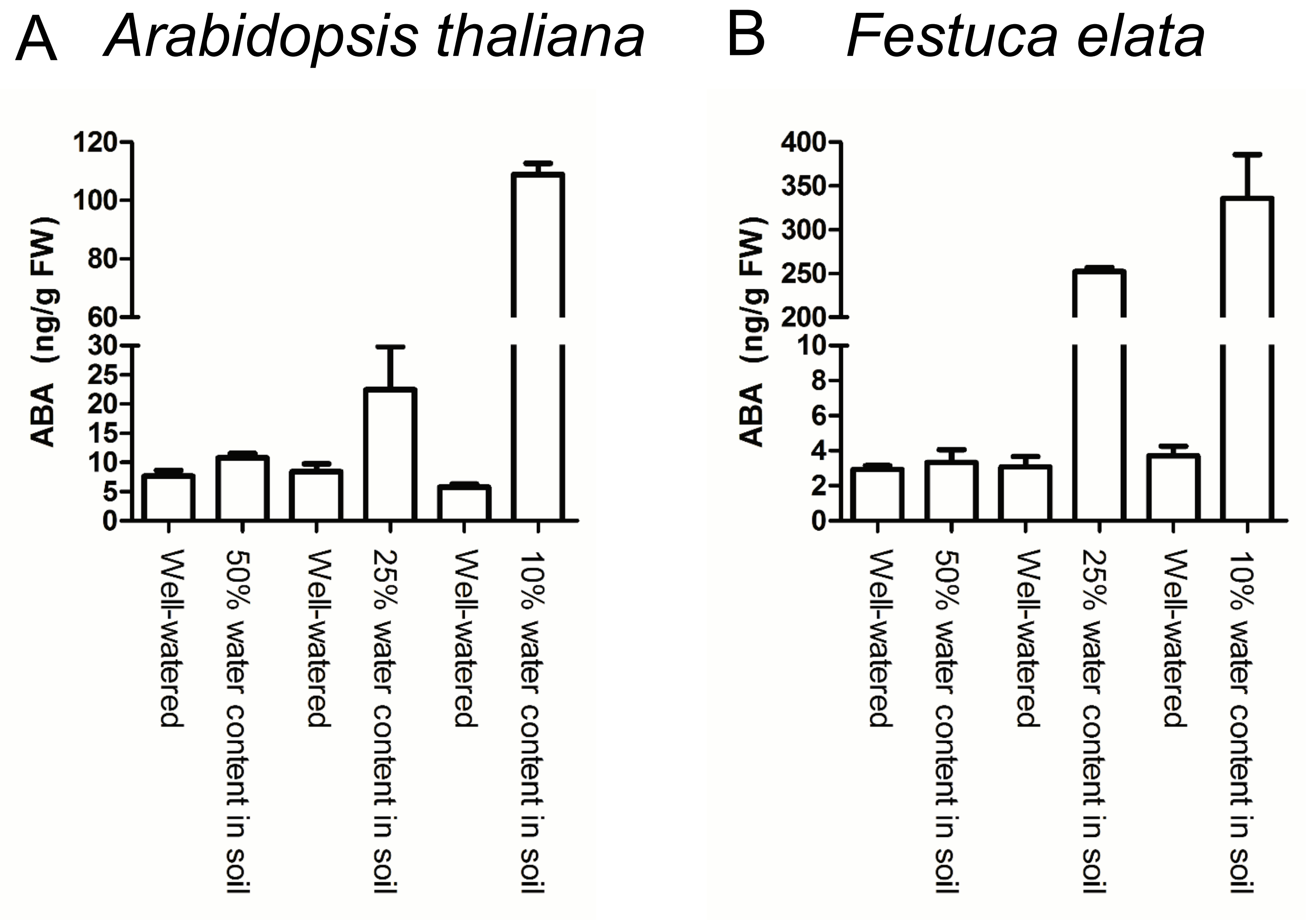

Supplemental Figure 5. Measurements of ABA contents in leaves

The ABA levels of leaves were measured by using UPLC-MS in 4-week-old *Arabidopsis thaliana* (Col-0) (A) and 1-week-old *Festuca elata* (B) under well-watered, and drought stress conditions (50%, 25%, and 10% water contents in soil). Error bar means ±SD of biological triplicates**.**

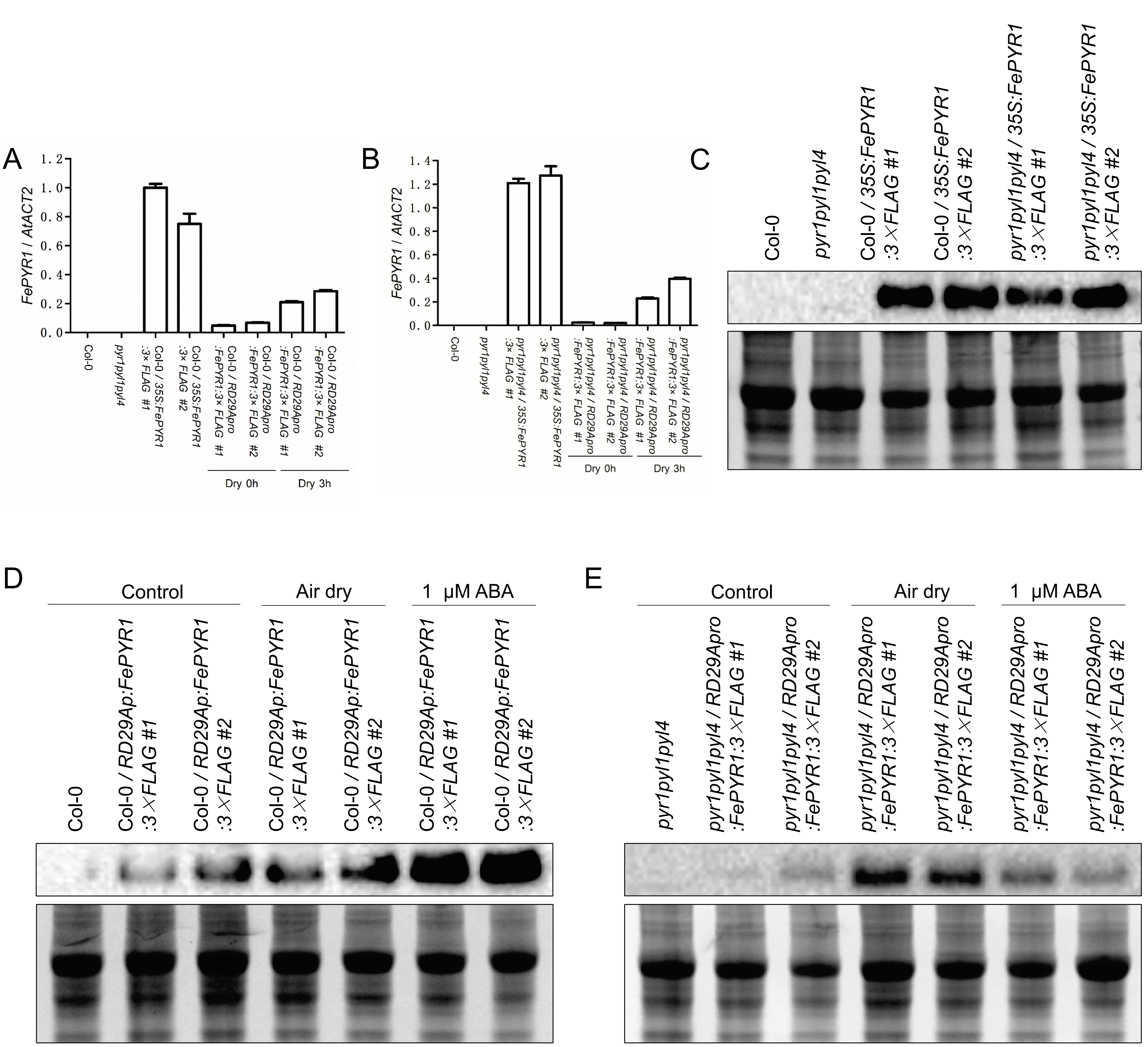

Supplemental Figure 6. FePYR1 expression in the transgenic plants.
(A, B) Quantitative measurement of *FePYR1* transcript levels in rosette leaves from 21-day-old Arabidopsis transgenic plants compared to *pyr1pyl1pyl4* and Col-0. Error bars represent ±SD of biological triplicates**.** (C - E) The protein levels of FePYR1-3×FLAG detected with anti-FLAG antibody in 21-day-old rosette leaves of *35S:FePYR1:3×FLAG* transgenic Col-0 and *pyr1pyl1pyl4* (C) , *RD29Apro:FePYR1:3×FLAG* transgenic Col-0 (D) and *pyr1pyl1pyl4* (E), with or without indicated treatments for 24 hours. Upper panel, FePYR1-3×FLAG protein. Lower panel, loading control (Coomassie Brilliant Blue staining). The full-length images for the blots and gels shown here were presented in Supplemental Figure 8, 9 and 10, respectively.

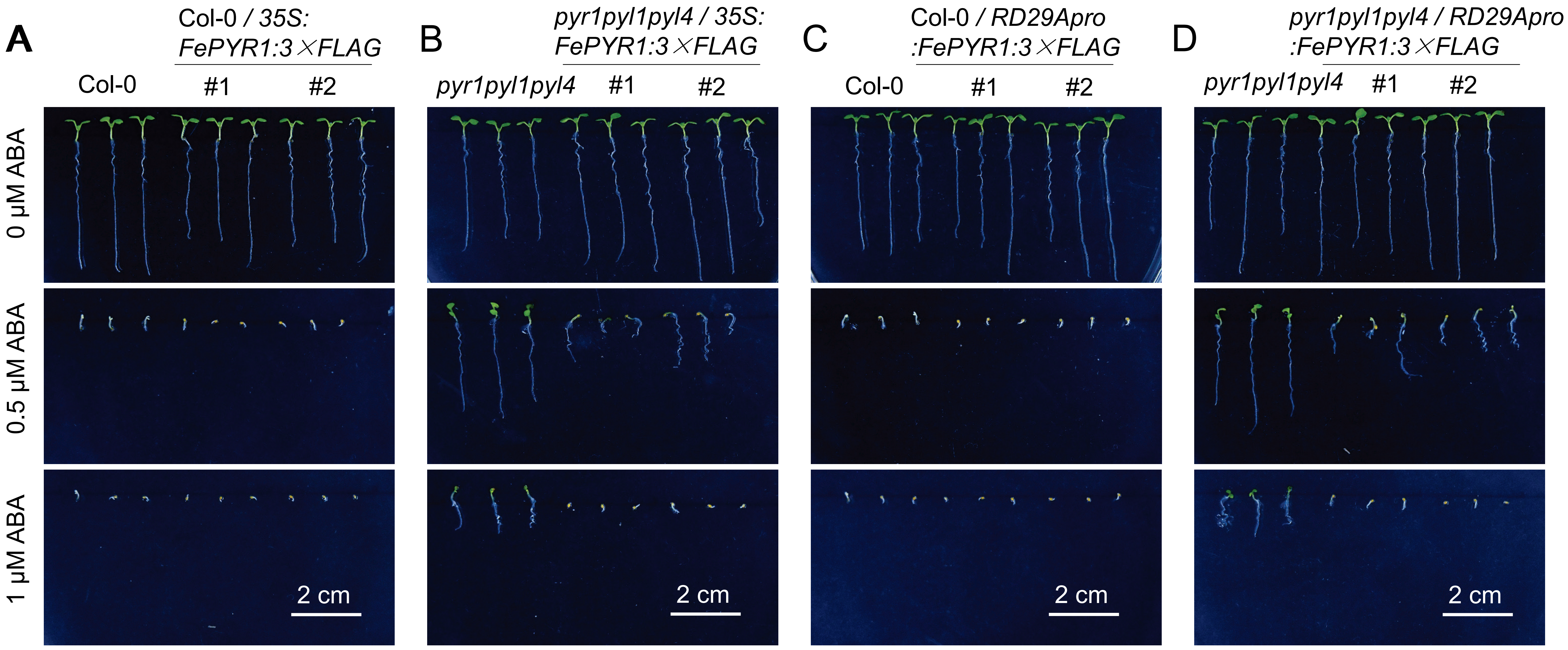

Supplemental Figure 7. FePYR1 partially rescues ABA insensitive phenotype of *pyr1pyl1pyl4* mutant during seed germination.

(A, B) Germination assay of wild type (Col-0), *pyr1pyl1pyl4*, and *35S:FePYR1:3×FLAG* transgenic plants grown in 0.5× MS medium containing 0, 0.5, or 1 μM ABA. The representative plants were photographed after growing for 7 days. Bar, 2 cm. (C, D) Seed germination assay of wild type (Col-0), *pyr1pyl1pyl4*, and *RD29Apro:FePYR1:3×FLAG* transgenic lines grown in 0.5× MS medium supplemented with 0, 0.5, or 1 μM ABA. The representative plants grown for 7 days before being photographed. Bar, 2cm.

A

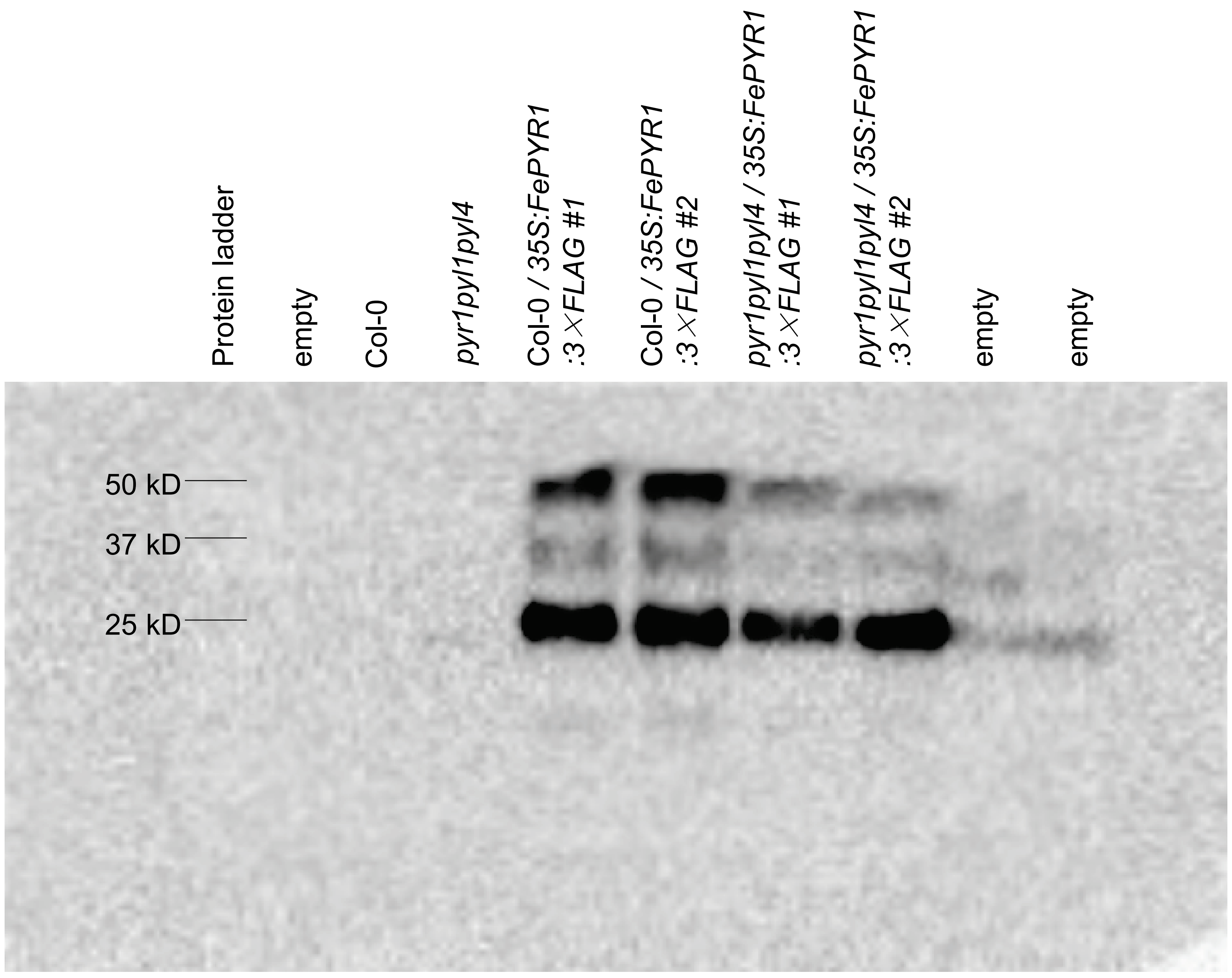

B

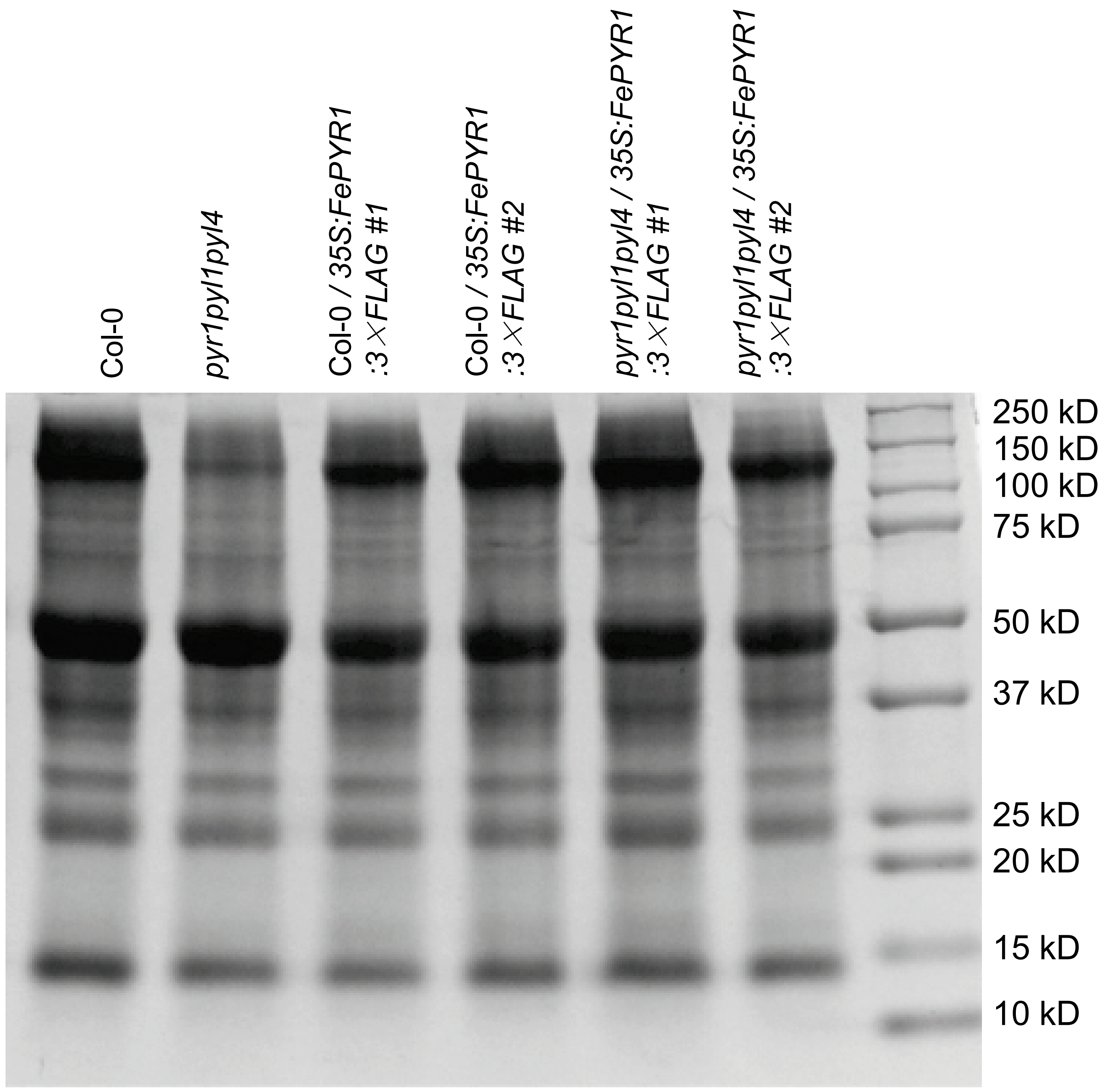

Supplemental Figure 8. The protein levels of FePYR1-3×FLAG detected in 21-day-old rosette leaves of *35S:FePYR1:3×FLAG* transgenic Col-0 and *pyr1pyl1pyl4*.

(A, B) The full-length blot(A) and gel(Coomassie Brilliant Blue staining)(B) images for Fig S6C. The images were generated by using ChemiDoc^{TM}XRS+ system in Image Lab^{TM} Software (Molecular Imager, *BIO-RAD*) with 60 seconds exposure time and bands auto-exposure mode, respectively. Empty means no sample loaded.

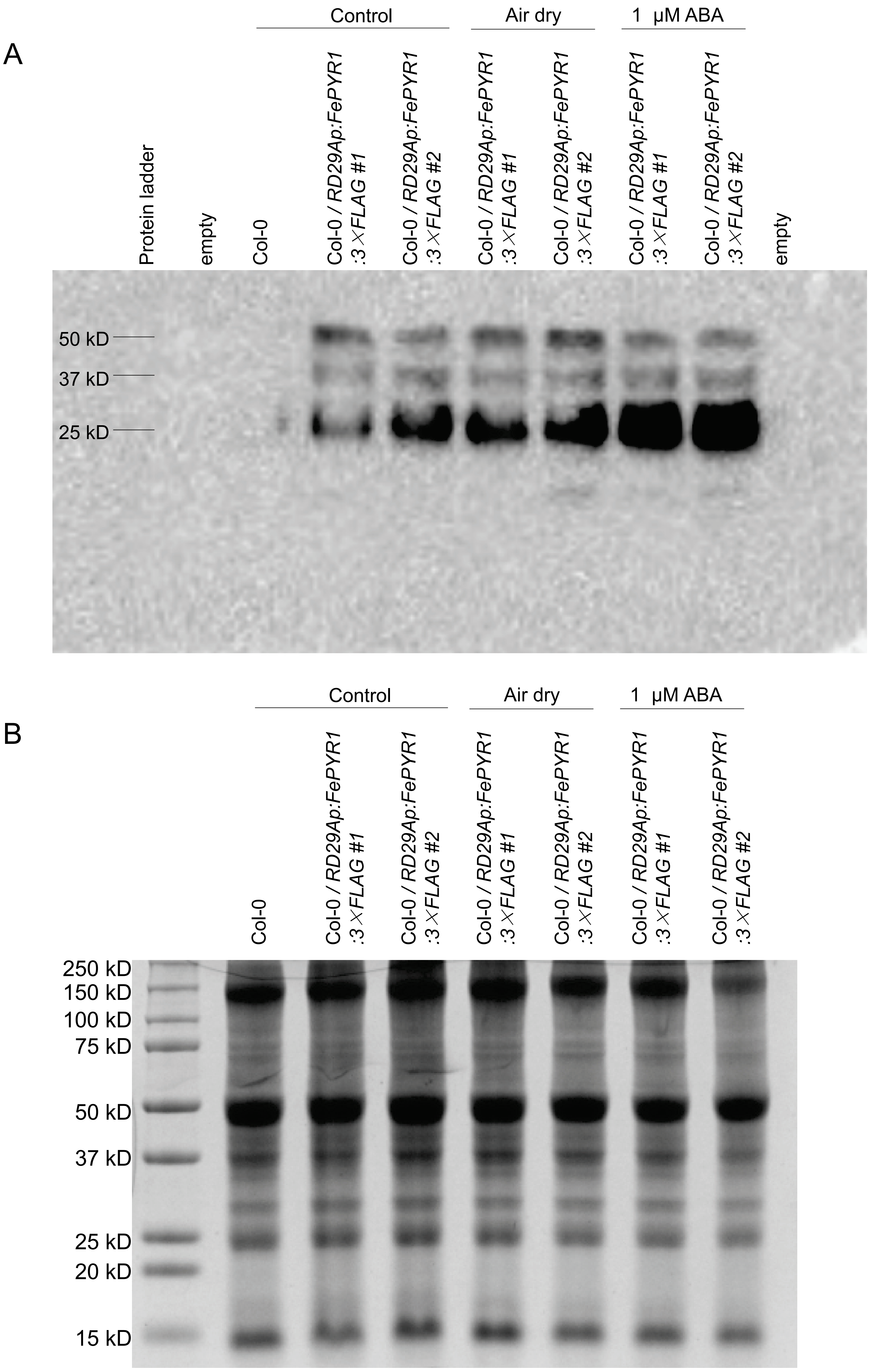

Supplemental Figure 9. The protein levels of FePYR1-3×FLAG detected in 21-day-old rosette leaves of *RD29Apro:FePYR1:3×FLAG* transgenic Col-0 plants

(A, B) The full-length blot(A) and gel(Coomassie Brilliant Blue staining)(B) images for Fig S6D. The plants were treated for 24 hours with or without indicated conditions. The images were generated by using ChemiDoc™XRS+ system in Image Lab™ Software (Molecular Imager, *BIO-RAD*) with 60 seconds exposure time and bands auto-exposure mode, respectively. Empty means no sample loaded.

A

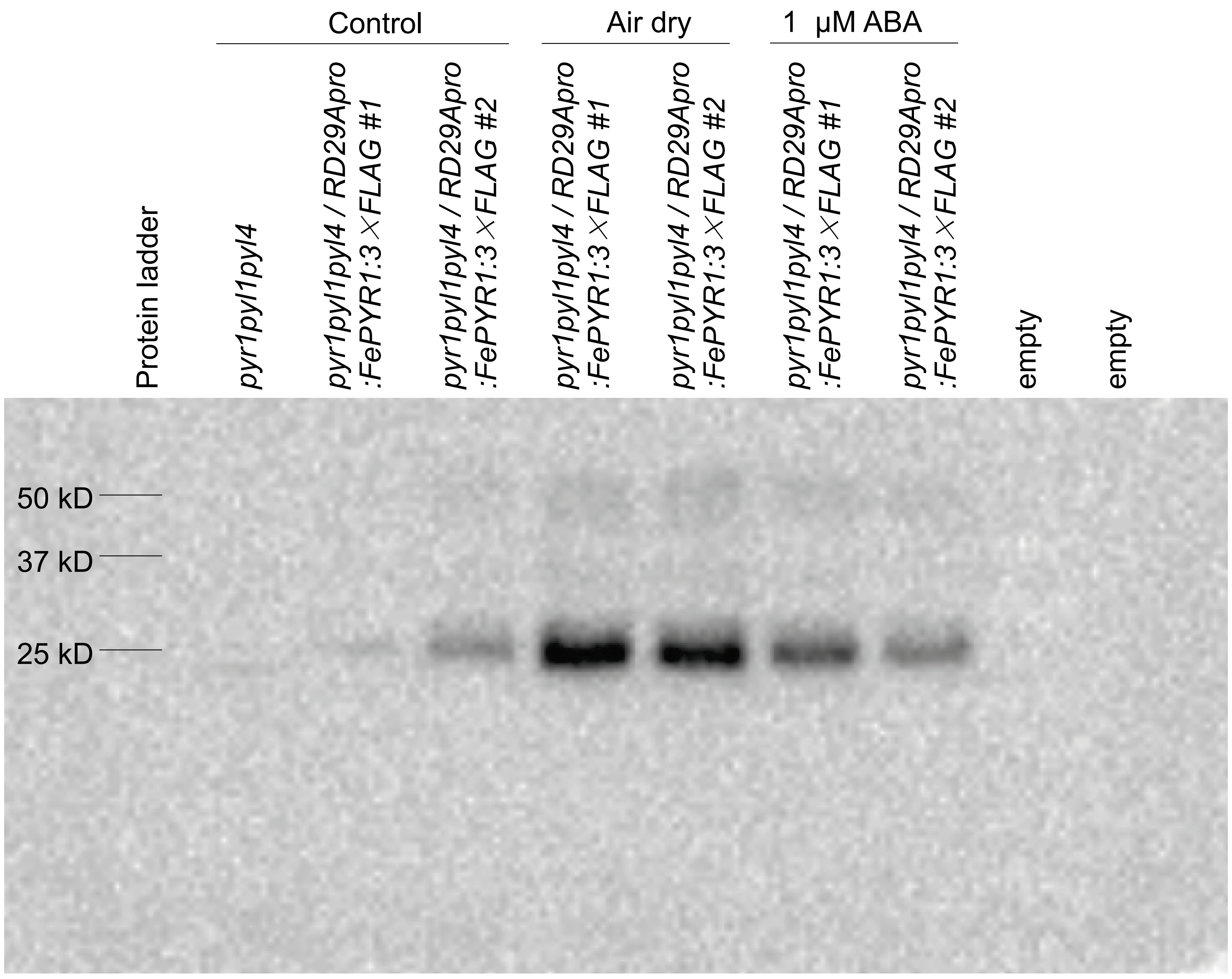

B

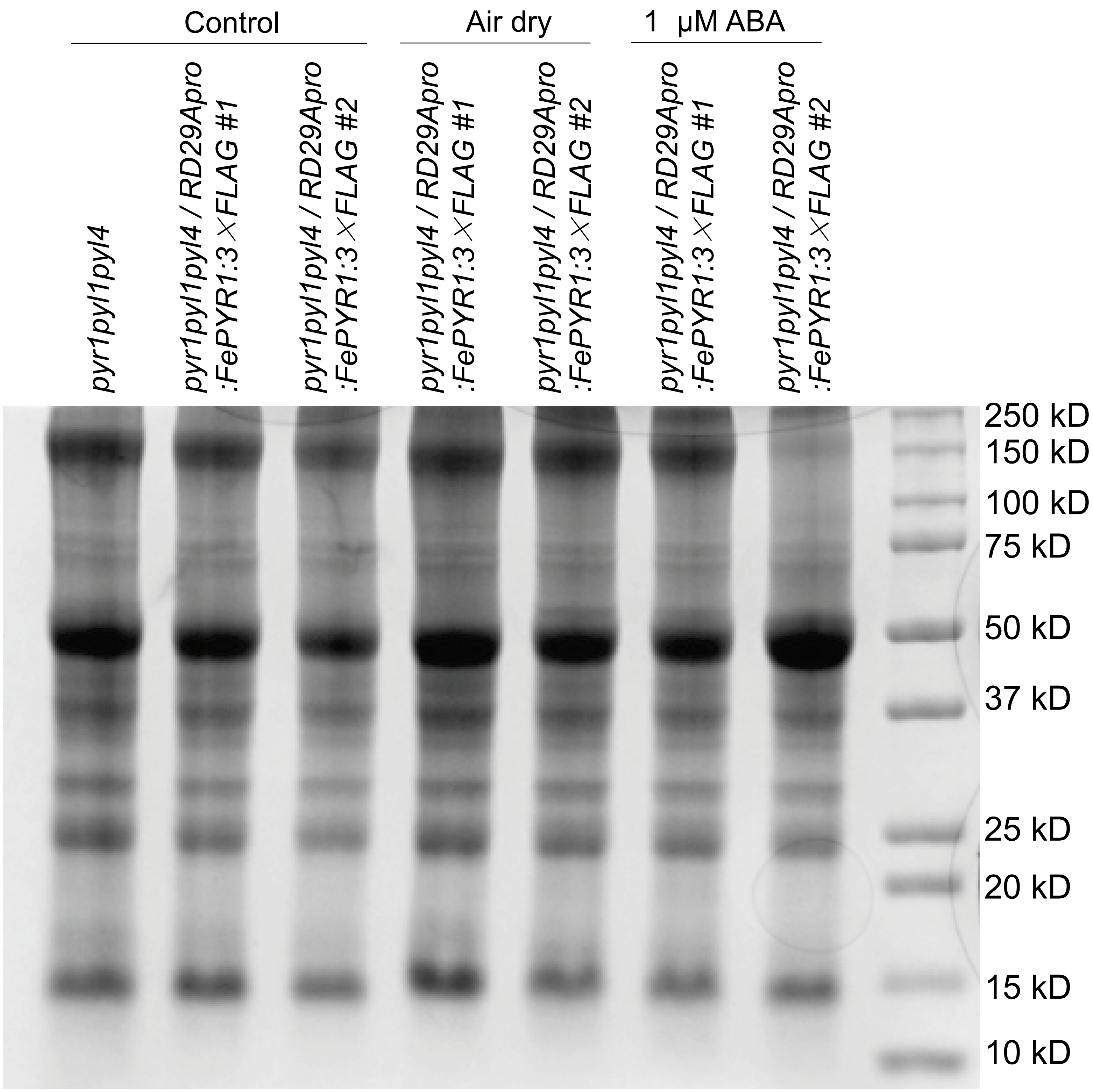

Supplemental Figure 10. The protein levels of FePYR1-3×FLAG detected in 21-day-old rosette leaves of *RD29Apro:FePYR1:3×FLAG* transgenic *pyr1pyl1pyl4* plants.

(A, B) The full-length blot(A) and gel(Coomassie Brilliant Blue staining)(B) images for Fig S6E. The plants were treated for 24 hours with or without indicated conditions. The images were generated by using ChemiDoc™XRS+ system in Image Lab™ Software (Molecular Imager, *BIO-RAD*) with 60 seconds exposure time and bands auto-exposure mode, respectively. Empty means no sample loaded.

Table S1 Statistics of data sets and structure refinement

**Table S1. Statistics of data sets and structure refinement**	
Space group	P 4_2 2_1 2
Resolution range, Å	41.3—2.7 (2.83-2.70)*
Cell parameters, Å, °	a=110.62, b=110.62,<br>c=124.2; α=β=γ=90
Total/Unique reflections	489348/20684
Completeness, %	96.3 (96.3)
Mean I/σ	11.7 (1.3)
Multiplicity	23.2 (21.9)
Rmerge	0.29 (3.84)
CC1/2	0.997 (0.306)
Refinement	
Resolution, Å	41.3—2.7
No. reflections	20636
No. residues	578
No.solvent molecules	39
No. of non-H atoms	4418
Rcryst	22.8%
Rfree	25.3%
rmsd bonds, Å	0.003
rmsd angles, °	0.87
Average B factor, Å2	87.0
*Values in the parentheses are for the highest resolution shell.	
